# Synergistic ROS/enzyme dual-responsive oral drug delivery system: A novel multi-mechanistic platform for spatiotemporal control and overcoming drug resistance in colorectal cancer therapy

**DOI:** 10.1016/j.mtbio.2025.101920

**Published:** 2025-05-30

**Authors:** Weitong Sun, Bingbing Fan, Xiaohan Qin, Xin Zhang, Pengxia Zhang, Yu Zhang

**Affiliations:** a College of Pharmacy, Jiamusi University, Jiamusi City, Heilongjiang Province, 154007, China; b Heilongjiang Pharmaceutical Research Institute, Jiamusi City, Heilongjiang Province, 154007, China

**Keywords:** Colorectal cancer, ROS/Enzyme dual-responsive, Drug resistance, Oral drug delivery, Synergistic therapy, Gut microbiota regulation

## Abstract

Colorectal cancer (CRC) remains a leading cause of cancer-related mortality, driven by complex interactions between inflammatory pathways, gut microbiota dysbiosis, and tumor microenvironment remodeling. Conventional therapies, particularly single-target oral chemotherapeutics, are hindered by poor bioavailability, systemic toxicity, and drug resistance. To address these limitations, we engineered KGM-PTX/CSM microspheres, a dual-responsive drug delivery system leveraging the elevated reactive oxygen species (ROS) in CRC and β-mannanase overexpression in the colorectum. The system comprises ROS-sensitive prodrug micelles (PSM) encapsulated within konjac glucomannan (KGM). PSM micelles were synthesized by conjugating hydrophilic chitosan oligosaccharides (COS) with the hydrophobic anti-inflammatory agent mesalazine (MSL) via ROS-labile thioether bonds, followed by paclitaxel (PTX) encapsulation. Upon oral administration, KGM undergoes β-mannanase-triggered degradation in the colon, releasing PSM micelles that subsequently disintegrate in the ROS-rich tumor microenvironment, enabling spatiotemporally controlled drug release. *In vitro* studies demonstrated ROS-responsive drug liberation (91.2 % cumulative release within 48 h) and enhanced cytotoxicity against PTX-resistant SW480/PTX cells (IC_50_: 9.33 μg/mL vs. 45.68 μg/mL for free PTX). Mechanistic investigations revealed synergistic interactions among the system's components: PTX stabilized microtubules to induce apoptosis, while MSL counteracted COX-2/P-gp-mediated drug resistance and alleviated PTX-associated intestinal inflammation. In the AOM/DSS-induced orthotopic CRC model, KGM-PTX/CSM significantly inhibited colorectal tumor growth, improved survival rates, and suppressed inflammatory cytokine expression (TNF-α, IL-1β, IL-6, and IL-10) in serum and colorectal tissues. Immunomodulatory effects included enhanced CD8^+^ T-cell activity, suppression of Treg-mediated immune evasion, and macrophage polarization toward the tumor-suppressive M1 phenotype. Gut microbiota analysis demonstrated restored operational taxonomic unit (OTU) counts, increased beneficial bacterial populations, elevated alpha and beta diversity, reduced pro-inflammatory bacteria, and increased short-chain fatty acid (acetate, propionate, and butyrate) concentrations, collectively improving intestinal microecology and inhibiting tumor progression. This study synergistically enhanced the anti-CRC effect through multiple mechanisms of action such as chemotherapy, reversal of chemotherapy resistance, regulation of intestinal flora, anti-inflammation, activation of immune cells, etc., which will provide a certain reference for the research of synergistic drug therapy for CRC.

## Graphical abstract

**Figure:**
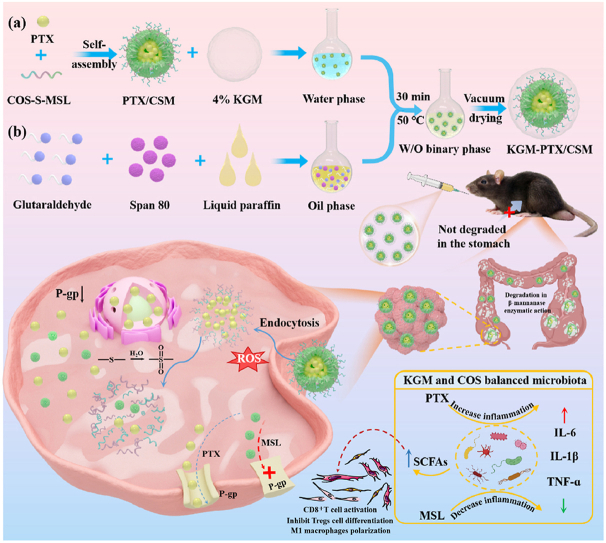

## 1 Introduction

Colorectal cancer (CRC), as a malignant tumor that seriously threatens human health worldwide, has a long history of high incidence and mortality [1,2]. According to the data released by the International Agency for Research on Cancer (IARC) in 2024, CRC has the third highest incidence rate and the fourth highest mortality rate of malignant tumors worldwide [3]. The occurrence of CRC may be influenced by complex microenvironments, including tumor microenvironment, chronic inflammation, intestinal microbiota disorder and immunosuppressive microenvironment [4,5]. Clinically, the disease often progresses asymptomatically, with approximately 60–70 % of cases diagnosed at advanced stages [6]. While surgical resection and radiotherapy provide localized tumor control, they exhibit limited efficacy against micrometastases and high recurrence rates. Systemic chemotherapy, particularly for metastatic CRC, serves as a cornerstone for managing disseminated malignancies but faces critical limitations [[7], [8], [9]]. Conventional oral chemotherapeutics encounter multiple gastrointestinal barriers, resulting in suboptimal pharmacokinetic profiles characterized by chemical instability, off-target distribution, and propensity for inducing drug resistance [10]. Furthermore, these agents frequently disrupt intestinal microbiota homeostasis and exacerbate inflammatory responses, potentially compromising therapeutic outcomes [11]. In view of these challenges, there is an urgent need to develop a synergistic drug delivery system with intelligent response release to achieve precise delivery and controlled release of drugs to multidimensionally enhance CRC therapeutic efficacy and reduce side effects.

Chitosan oligosaccharides (COS), a class of natural polymer-derived materials, are enzymatically hydrolyzed products of chitin. Characterized by excellent water solubility and permeability, this biomaterial exhibits remarkable application potential in the biomedical field owing to its outstanding biocompatibility and biodegradability [12,13]. Their unique physicochemical properties enable applications in nanocarrier systems (e.g., polymeric micelles, nanoparticles) while demonstrating dual functionality as both therapeutic agents and delivery vehicles [14]. Mechanistically, COS exerts multimodal antitumor effects through: (i) antimicrobial activity against pathogenic Enterobacteriaceae, (ii) antioxidant-mediated reduction of pro-inflammatory cytokines, and (iii) immunomodulation via dendritic cell activation. Notably, COS enhances intestinal short-chain fatty acid production (e.g., acetate) while promoting beneficial bacterial populations like Lactobacillus, thereby restoring microbiota equilibrium and potentiating chemotherapeutic efficacy through gut-tumor axis modulation [15,16].

Paclitaxel (PTX), a microtubule-stabilizing agent derived from Taxus species, demonstrates significant therapeutic effects in CRC [17,18]. Its antimitotic mechanism involves GTP-independent tubulin polymerization arrest, inducing G2/M phase cell cycle blockade and caspase-mediated apoptosis [19,20]. However, PTX administration disrupts colorectal microbiota homeostasis by reducing Bifidobacterium abundance and compromising mucin-2-dependent barrier integrity, triggering IL-6/IL-1β-driven inflammation [21,22]. Compounding these limitations, CRC cells frequently overexpress P-glycoprotein (P-gp) efflux pumps, conferring multidrug resistance (MDR) through ATP-dependent PTX extrusion [23]. These factors limit its further wide application in CRC. Therefore, searching for effective strategies to overcome the challenges of PTX in CRC treatment has become a hot topic in clinical application [24]. Mesalazine (5-ASA), a frontline ulcerative colitis therapeutic, counteracts these challenges via dual anti-inflammatory/chemosensitization mechanisms [25]. It suppresses prostaglandin synthesis through COX-2 inhibition (IC_50_: 3.2 μM) while disrupting NF-κB/Wnt crosstalk to attenuate CRC progression [26,27]. Critically, MSL downregulates COX-2-mediated P-gp phosphorylation, reversing PTX resistance in MDR CRC models [28,29]. However, both PTX and MSL have the disadvantages of poor water solubility, so it is necessary to design an intelligent drug delivery system to increase the solubility of PTX and MSL, reduce drug toxicity, reverse drug resistance and enhance their synergistic anti-tumor effect against CRC.

In recent years, the development of prodrug nanotechnology has made it possible to combine therapeutic agents with tumor-responsive carriers. In particular, polymer-based prodrug micelle systems can improve drug stability and loading capacity, enhance the solubility of hydrophobic drugs through their core-shell structure [[30], [31], [32], [33]]. And leverage the characteristics of the tumor microenvironment (e.g., pH, ROS, GSH) to achieve targeted activation [34,35]. However, when administered orally, these nano-systems face multiple challenges in the gastrointestinal tract, such as gastric acid, digestive enzymes, and the complex gut microbiota, which may lead to premature drug release or carrier degradation, compromising delivery efficiency and therapeutic efficacy. Therefore, the development of intelligent delivery systems with oral stability is of great significance for achieving precise treatment of CRC [[36], [37], [38], [39]]. Konjac glucomannan (KGM), as an ideal colon-targeting carrier, exhibits excellent biocompatibility and biodegradability. It can be enzymatically degraded by β-mannanase, enabling precise drug release in the colorectal region [[40], [41], [42], [43]]. Additionally, KGM promotes the growth of beneficial bacteria and improves intestinal barrier function, further enhancing its anti-inflammatory and immunomodulatory effects. Based on KGM, intelligent delivery systems are expected to overcome the limitations of traditional oral nano-systems, improving the precision of drug delivery and facilitating synergistic therapy for CRC.

**Fig. 1:**
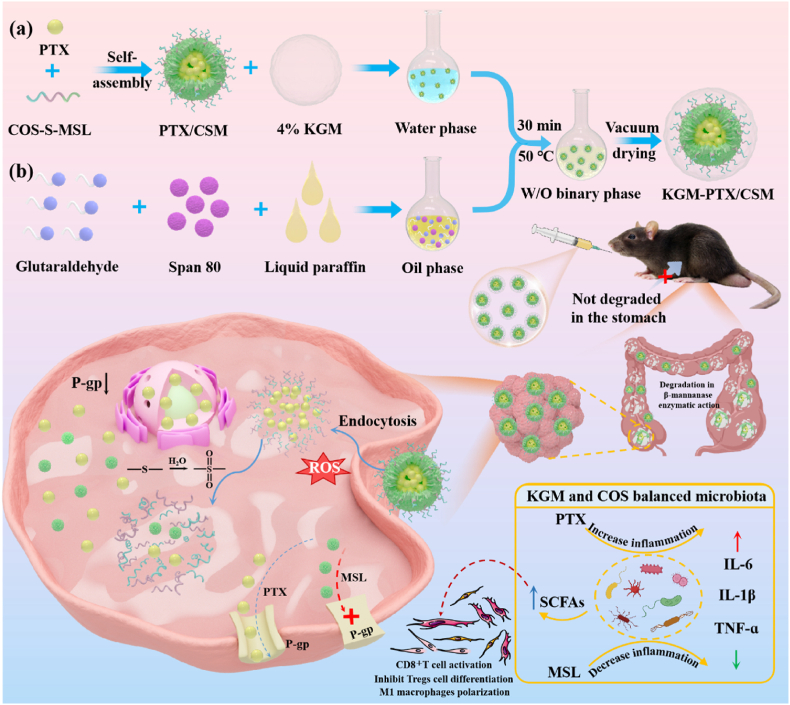
Preparation and therapeutic process of KGM-PTX/CSM.

Based on this, a ROS/enzyme dual-responsive smart delivery system was constructed in this study, and KGM-PTX/CSM microspheres were prepared by loading PTX with MSL into COS-based pre-drug polymer CSM micelles and utilizing KGM encapsulation. The drug system was designed to achieve precise delivery and synergistic treatment of CRC through β-mannanase-mediated colon-targeted delivery and drug release triggered by high levels of ROS in tumor cells, playing a dual-function role of drug/carrier, and synergistically enhancing the effect of anti-CRC through multiple mechanisms of action, such as chemotherapy, reversal of drug resistance, regulation of intestinal flora, anti-inflammation, activation of immune cells, etc., which provided a new way for the research of synergistic multi-targeted drug therapy for CRC. It provided a new way for CRC synergistic multi-target drug therapy research. Preparation and therapeutic process of KGM-PTX/CSM is shown in Fig. 1.

## 2 Materials and methods

### 2.1 Materials

Paclitaxel(33069-62-4, >99 %), Chitosan oligosaccharide and mesalazine were purchased from Merck Life Science Co, Ltd. Konjac glucomannan (KGM) was obtained from Hefei Pomeranian Biological Company. Dialysis bag (MWCO 3500 Da) was purchased from Shanghai Source Leaf Biological Co., Ltd. (Shanghai, China). Thiodiacetic acid, pyrene, 1-Ethyl-(3-dimethylaminopropyl) carbodiimide (EDC), were purchased from Aladdin Reagent Co., Ltd. (Shanghai, China). N-Hydroxysuccinimide (NHS) was purchased from Shanghai Maclin Biochemical Technology Co., Ltd. (Shanghai, China). 4-Dimethylaminopyridine (DMAP) was purchased from Wuhan Huazhu Medical Technology Co., Ltd. (Wuhan, China). Penicillin-streptomycin solution was obtained from Zhejiang Tianhang Biotechnology Co., Ltd. (Zhejiang, China). Fetal bovine serum, Trypsin digestion solution, DAPI staining solution, Cell lysis buffer (RIPA), Hematoxylin staining solution and Eosin staining solution were acquired from Biyuntian Biotechnology Co., Ltd. 96-well cell culture plate and 6-well cell culture plate were bought from Costar, USA. 4 % paraformaldehyde was presented by Beijing Lanjieke Technology Co., Ltd. (Beijing, China). CY5 was presented by AkangTrading Co., Ltd (Shanghai, China). BCA protein quantification kit was presented by Kangwei Century Biotechnology Co., Ltd. Rabbit anti-human anti-MRP2 antibody and Goat anti-rabbit horseradish peroxidase-labeled secondary antibody were obtained from Invitrogen, USA. Interleukin-6 (IL-6) kit, Tumor necrosis factor (TNF-α) kit, Interleukin-13 (IL-1B) kit were acquired from Nanjing Jiancheng Technology Co., Ltd.

### 2.2 Synthesis and characterization CSM and CM

#### 2.2.1 Synthesis of the TDAA

3 g of TDA was weighed and dissolved in 9 mL of acetyl chloride (TDA:acetyl chloride molar ratio = 1:12), and the solution was heated to 65 °C and refluxed for 2 h. After the reaction, most of the acetyl chloride was removed by rotary evaporation (40 °C, 0.1 MPa), and the precipitated product was added to the excess ice ether ice bath. The product was extracted and dried in a vacuum-dried at 40 °C for 24 h to obtain TDAA.

#### 2.2.2 The synthesis of COS-S

A certain amount of COS, TDAA, EDC and NHS (molar ratio COS: TDAA: EDC: NHS = 1:1.2:1.5:1.5) were weighed and dissolved in an appropriate amount of DMSO (10 mL per 100 mg COS), and after reaction at room temperature for 48 h, the reaction solution was transferred to a dialysis bag (MV = 3500 Da) and dialysis was performed with purified water for 48 h to remove small molecular impurities. The product COS-S was freeze-dried and stored at −20 °C.

#### 2.2.3 Synthesis of COS-S-MSL

COS-S, EDC, and NHS (molar ratio COS-S: EDC: NHS = 1:1.2:1.2) were dissolved in DMSO and stirred for 2 h to activate carboxyl groups. MSL was added (molar ratio COS-S:MSL = 1:0.8) and reacted for 48 h under nitrogen. The product was dialyzed as described in 2.2.2 and lyophilized.

#### 2.2.4 Synthesis of COS-MSL

An appropriate amount of COS, MSL, EDC and NHS (molar ratio COS: MSL: EDC: NHS = 1:1:1.5:1.5) were weighed and dissolved in DMSO, and the reaction was carried out for 48 h at room temperature. The product solution was then dialyzed with purified water for 2 d and lyophilized to obtain the COS-MSL.

#### 2.2.5 Characterization of CSM and CM

To ascertain the successful synthesis of CSM, structural analysis was conducted using both ^1^H NMR spectroscopy (D_2_O, 400 MHz) and FT-IR techniques (4000-400 cm^−1^, KBr pellet method). Spectra were analyzed using MestReNova v14.0 and OMNIC v9.2 software, respectively.

### 2.3 Determination of critical aggregation concentration (CAC)

A CSM stock solution (1 mg/mL) was diluted to concentrations ranging from 0.001 to 1 mg/mL. Pyrene solution (1 × 10^−6^ M in acetone) was added (20 μL per sample). After 24 h equilibration in the dark, fluorescence intensities at 373 nm (I_1_) and 384 nm (I_3_) were measured using a Hitachi F-7000 spectrofluorometer (slit width: 5 nm, integration time: 1 s). The CAC was determined from the inflection point of the *I*_*1*_*/I*_*3*_ vs log *C* plot.

### 2.4 Synergistic effect of PTX and MSL

To determine the optimal synergistic ratio of PTX and MSL in the treatment of colorectal cancer, the CCK-8 assay was employed. SW480/PTX cells cultured in RPMI-1640 medium supplemented with 10 % FBS were seeded into 96-well plates at a density of 1 × 10^4^ cells per well and incubated for 12 h (37 °C, 5 % CO_2_). when the cell confluence reached 70–80 %, different concentrations of drug solutions (PTX:MSL ratios of 4:1, 2:1, 1:1, 1:2, and 1:4) were added to the culture medium. After 48 h incubation, 10 μL of CCK-8 reagent was added to each well, followed by a 2 h incubation in the dark. Absorbance at 450 nm was measured using a BioTek Synergy H1 microplate reader, with blank wells containing medium only. The cell inhibition rate was calculated using the following formula: Inhibition Rate (%) = [(OD_control_ - OD_sample_)/(OD_control_ - OD_blank_)] × 100 %. IC_50_ values were derived via nonlinear regression (GraphPad Prism v10.0), and combination indices (CI) were calculated using the Chou-Talalay method (CalcuSyn v2.1). Synergism was defined as CI < 1.

### 2.5 Preparation and characterization of PTX/CSM micelles

PTX/CSM micelles were fabricated using a solvent evaporation technique. Aqueous micellar solutions were prepared by dissolving 20 mg of CSM polymer in 10 mL purified water under continuous magnetic stirring (500 rpm). Concurrently, 3 mg of paclitaxel (PTX) was dissolved in 5 mL methanol via probe sonication (40 kHz, 100 W, 10 min). The PTX-methanol solution was introduced dropwise (0.5 mL/min) into the CSM solution under ambient conditions (25 °C), followed by 4 h of vigorous stirring. The resulting colloidal dispersion was filtered through a 0.22 μm polyvinylidene fluoride (PVDF) membrane to remove unencapsulated drug aggregates.

The physicochemical properties of the micelles were systematically characterized. Dynamic light scattering (DLS, Malvern Zetasizer Nano ZS) measurements at 25 °C provided hydrodynamic diameter, polydispersity index (PDI), and zeta potential values, with triplicate analyses performed for statistical reliability. Morphological evaluation was conducted via transmission electron microscopy (TEM, Hitachi HT7700) after negative staining with 2 % uranyl acetate (120 kV accelerating voltage). The PTX, CSM, physical mixture (PTX + CSM), and PTX/CSM were respectively mixed and ground with potassium bromide (KBr), then compressed into tablets and scanned using a Fourier transform infrared spectrometer. DSC scans were performed on the physical mixture (PTX + CSM), PTX/CSM micelles, PTX, and CSM samples, and the results were shown in Fig. 7D. The characteristic absorption peaks of PTX and CSM were 279.1 °C and 82.7 °C, respectively, which do not overlap with the characteristic peaks of PTX. The characteristic peaks of CSM and PTX appeared on the heat curves of the physical mixture, while only the characteristic peaks of CSM appeared on the heat curves of PTX/CSM micelles, suggesting that PTX was encapsulated into a carrier and existed in an amorphous form, which further indicated the PTX/CSM micelles were successfully prepared. Drug loading capacity (DL%) and encapsulation efficiency (EE%) were quantified using ultracentrifugation (7000×*g*, 30 min, 4 °C) to separate free PTX, followed by HPLC analysis (Agilent 1260 system equipped with a C18 column). The mobile phase consisted of acetonitrile and water (70:30 v/v) delivered at 1.0 mL/min. Calculations were performed as follows:

DL(%)=(weightofdruginmicelles/weightofPTX/CSMmicelles)×100%

EE(%)=(weightofdruginmicelles/initialweightofdrug)×100%

### 2.6 Stability tests

#### 2.6.1 Dilution stability

1 mL of the PTX/CSM micelles was aliquoted and diluted with purified water at ratios of 0, 5, 10, 20, and 50, respectively. Subsequently, the particle size and encapsulation efficiency of the diluent were determined and the results were analyzed.

#### 2.6.2 Storage stability

The PTX/CSM micelles were placed at 4 and 25 °C for 30 days, and the particle size and encapsulation efficiency of the micelles were determined at 1, 5, 7, 15, 30 days, respectively, and the results were analyzed.

#### 2.6.3 Stability in plasma

An appropriate amount of PTX/CSM micelles were placed in PBS scontaining 10 % fetal bovine serum (v/v) at 37 °C for 24 h. Samples were taken at 0, 6, 12, 24, and 48 h, and the particle size and encapsulation efficiency were measured. Then the obtained results were analyzed.

### 2.7 Hemolysis test

Fresh mouse blood collected in heparin-coated tubes was centrifuged (3000×*g*, 10 min, 4 °C). Erythrocytes were washed three times with 0.9 % saline and resuspended to prepare a 2 % (v/v) RBC suspension.

For hemolysis testing, 1 mL of RBC suspension was mixed with 3 mL of PTX/CSM solutions at concentrations of 2–32 mg/mL. Negative controls (3 mL saline) and positive controls (3 mL deionized water) were processed in parallel. After 4 h incubation (37 °C), samples were centrifuged (3000×*g*, 10 min) and supernatants analyzed spectrophotometrically at 450 nm. Hemolysis percentage was calculated as:

Hemolysisrate(%)=(Asample−Anegative)/(Apositive−A)negative×100%

### 2.8 In vitro drug release of PTX/CSM micelles and PTX/CM micelles

To validate the ROS-responsive behavior of PTX/CSM micelles, samples (1 mL) were incubated in pH 7.4 PBS with or without 10 mM H_2_O_2_ at 37 ± 0.5 °C under gentle agitation (50 rpm). Hydrodynamic diameter changes were monitored by DLS (Malvern Zetasizer Nano ZS) at 0, 1, 5, 8, 12, and 24 h. TEM (Hitachi HT7700) was used to observe morphological alterations. For drug release studies, 2 mL of PTX solution, MSL solution, PTX/CM micelles, and PTX/CSM micelles were loaded into dialysis bags (MWCO 3500 Da). PTX and MSL solutions were dialyzed against pH 7.4 PBS with 0.5 % Tween-80, while micelles were dialyzed in PBS with or without 10 mM H_2_O_2_. Release experiments were conducted at 37 ± 0.5 °C (100 rpm). Aliquots were collected at 0.5–48 h, replaced with fresh medium, and analyzed by HPLC to calculate cumulative release (%).

### 2.9 Culture and drug resistance determination of SW480/PTX cells

SW480 human colorectal cancer cells were cultured in RPMI-1640 supplemented with 10 % FBS. Resistance induction was performed via stepwise PTX exposure: cells were treated with 0.1 μg/mL PTX until stable growth, followed by incremental increases to 25.0 μg/mL over 10 passages. Resistance index (RI) was calculated as:

RI = IC_50_ (SW480/PTX)/IC_50_ (SW480), when the RI > 5, the drug resistant cell culture was completed.

### 2.10 Cell uptake

SW480/PTX cells were seeded in 6-well plates (5 × 10^4^ cells/well) and incubated for 24 h. Media were replaced with PTX/CM or PTX/CSM micelles (PTX equivalent: 10 μg/mL). After 1.5 h incubation, cells were washed with PBS, fixed with 2.5 % paraformaldehyde, and stained with DAPI (1 μg/mL, 2 min, dark). Cellular uptake was visualized using a Leica DMi8 fluorescence microscope (20 × objective).

### 2.11 Cell cytotoxicity

#### 2.11.1 Cell cytotoxicity of blank carriers CM and CSM

The cytotoxicity of the blank carriers CSM and CM was evaluated on SW480 and SW480/PTX cells using the CCK-8 assay method. The original medium was discarded, and fresh medium containing the blank carriers of CM and CSM was added, with five concentration gradients set for each (1, 10, 35, 70, 100 μg/mL). After sealing the well plates and incubating for 48 h, 10 μL of CCK-8 solution was added, followed by an additional incubation period of 2 h. Cell viability was then calculated using the following formula:

$$\text{Cell}\;\text{viability}\%=\left({\text{OD}}_{\text{sample}}-{\text{OD}}_{\text{blank}}\right)/\left({\text{OD}}_{\text{control}}-{\text{OD}}_{\text{blank}}\right)\times 100\%$$

#### 2.11.2 Cell cytotoxicity of drug-loaded micelles PTX/CM and PTX/CSM

The experiment comprised four groups: free PTX, PTX + MSL (1:2 M ratio), PTX/CM micelles, and PTX/CSM micelles, with concentrations of PTX in each group set at 1, 2, 4, 12, 24, and 40 μg/mL, respectively. All other procedures and calculation formulas follow those outlined in Section "3.1.1".

### 2.12 Cell migration assay

The inhibitory effects of MSL, PTX, MSL + PTX, CM, CSM, PTX/CM micelles and PTX/CSM micelles on SW480/PTX cells migration were investigated by cell scratch assay. SW480/PTX cells were inoculated into a 6-well plate and grown to near confluency. Then scratches were created within the cell layer using the tip of a sterile pipette gun, washed with PBS and added the medicated medium. Images of different groups were taken at 0 and 48 h using fluorescent inverted microscopy, with the untreated cells used as controls.

### 2.13 Western blot assay

Proteins were abstracted from SW480/PTX cells using RIPA lysis buffer to quantify protein concentrations by a BCA protein assay. After the protein concentrations were determined, proteins were boiled in PBS with a loading buffer. The samples were separated by 12 % SDS-PAGE gel electrophoresis and transferred to PVDF membranes (Millipore, USA). The membrane was wetted with PBST, and the corresponding bands were cut according to the molecular weight of the protein and placed in 5 % skim milk to close the membrane. Then, the membrane incubated with the primary antibody (1:1000, BIOSS, USA). Subsequently, the membrane was washed three times, and then incubated with horseradish peroxidase conjugated goat anti-rabbit IgG secondary antibodies (1:5000, Invitrogen, USA). Protein bands were visualized by the enhanced chemiluminescence (ECL) reagent.

### 2.14 Preparation and characterization of KGM-PTX/CSM microspheres

#### 2.14.1 Preparation of KGM-PTX/CSM microspheres

KGM-PTX/CSM microspheres were prepared via emulsification-crosslinking. Briefly, 0.05 g PTX/CSM micelles were dispersed in 4 % konjac glucomannan (KGM) solution (w/v) as the aqueous phase (W). Separately, 1 mL Span-80 was homogenized into 50 mL liquid paraffin (oil phase, O). The KGM solution was injected into the oil phase via a 22G syringe needle under mechanical stirring (500 rpm, 50 °C), forming a water-in-oil (W/O) emulsion. After 30 min stabilization, 0.5 mL 2 % glutaraldehyde was added as a crosslinker. The mixture was stirred for 2 h at 50 °C, followed by centrifugation (8000×*g*, 10 min) to collect microspheres. Purification involved sequential washing with petroleum ether and deionized water (3 × each). The final product was vacuum-dried (40 °C, 24 h).

#### 2.14.2 Characterization of KGM-PTX/CSM microspheres

The morphology of KGM-PTX/CSM microspheres was observed using a Regulus 810 Field Emission Scanning Electron Microscope (Hitachi Science Instruments Ltd., Beijing, China). The particle size was determined through Dynamic Light Scattering (DLS, Nano-s Ltd., Malvern Instruments, UK). The fourier transform infrared spectra of KGM-PTX/CSM microspheres was recorded using Spectrum 100 FT-IR spectrometer (PerkinElmer Ltd., Shanghai, China). The thermal behavior of PTX/CSM micelles, KGM, physical mixtures PTX/CSM + KGM and KGM-PTX/CSM microspheres were analyzed by thermos gravimetric analyzer (DSC, STA409PC, Hengjiu Ltd., Xuzhou, China).

The peak area of the sample was measured by HPLC system and the concentration of PTX was calculated. DL% and EE% of the PTX in KGM-PTX/CSM microspheres were calculated using the following formulas:

DL%=(weightofPTXinmicelles/weightofKGM−PTX/CSM)×100%

EE%=(weightofPTXinmicelles/weightofPTXaddedduringpreparation)×100%.

### 2.15 ROS and enzyme dual-responsive drug release

The Controlled Release Behavior and ROS and enzyme dual targeting of KGM-PTX/CSM microspheres were evaluated by simulating the *in vitro* release environment. Briefly, 20 mg of KGM-PTX/CSM microspheres was mixed with 2 mL of pH7.4 PBS in a dialysis bag, which was then placed in an artificial gastric fluid of pH1.2 and shaken at 100 rpm at a constant temperature of 37 °C for 2 h. After that, the release medium was sequentially adjusted to pH 6.8 artificial small intestine fluid and artificial colon fluid (pH 7.4 PBS solution + 0.5 % Tween-80 + 10 mM H_2_O_2_ + 0.2 U/mL β-mannanase) and incubated for 3 h, respectively. A sample of 3 mL was taken in a centrifuge tube at regular intervals and replenished with an equal volume of release medium. The peak areas of PTX and MSL were measured by HPLC after the samples were permeated, and the cumulative release rate was calculated and the *in vitro* release curves of KGM-PTX/CSM microspheres were plotted.

### 2.16 Gastrointestinal distribution experiment

#### 2.16.1 Preparation of IR780 iodide-labeled microspheres

IR780 iodide (3 mg) was dissolved in 300 mg blank self-emulsion via probe sonication (40 kHz, 10 min). The emulsion was mixed with KGM-PTX/CSM microspheres and crosslinked with 1 % CaCl_2_ under high-voltage electrostatics (15 kV, 30 min).

#### 2.16.2 Gastrointestinal distribution of IR780 iodide-labeled microspheres

A SPF-grade male C57BL/6J mouse (20 ± 2 g) was orally administered IR780-labeled microspheres. After 6 h, mice were sacrificed and gastrointestinal tissues were removed to detect the fluorescence distribution of microspheres in the gastrointestinal tract.

### 2.17 In vivo antitumor activity

#### 2.17.1 Mice model and treatment

In this work, male C57BL/6 mice of SPF grade, weighing between 20 and 22 g, and aged 4–6 weeks, were selected. These mice were procured from Liaoning Changsheng Biotechnology Co., Ltd. (Certification No.: SCXK (Liao) 2020-0001) (Ethics No JMSU-2023110803). They were housed in an SPF environment maintained at a temperature of 23 ± 2 °C and an average humidity of 58 ± 5 %. Ad libitum access to drinking water and feed was provided, and the animals were allowed an acclimatization period of one week. Both animal husbandry practices and experimental procedures adhered strictly to the ethical guidelines for animal experimentation at Jiamusi University.

The mice were randomly assigned into seven groups, namely the blank control group, the model group, the COS group, the PTX + MSL group, the PTX/CM micelles group, the PTX/CSM micelles group, and the KGM-PTX/CSM microspheres group, with 10 mice in each group. The mice model of CRC was established by using the AOM/DSS chemoinduction assay (Fig. 8B), and mice in each modeling group were subjected to an intraperitoneal injection of 10 mg/kg AOM on the initial day of experimentation. One week thereafter, they were provided with free access to 2 % DSS solution for 7 days, followed by 14 days of normal water consumption. This 21-day period constituted one cycle, and the modeling process was completed after three cycles. Upon successful modeling, each treatment group was administered drugs via intravenous injection at a dose of 10 mg/kg every 2 days for a period of 30 days. Throughout the experiment, mice were monitored for changes in fur color, activity levels, mental status, feces consistency, and anal area, while their body weight was measured and recorded weekly. All experimental mice were sacrificed after 30 days of intervention and the colon tissues of mice were collected for further analysis.

#### 2.17.2 Histology analysis

In order to perform histopathological analysis, the colon tissues were removed and preserved in 4 % paraformaldehyde solution to prepare hematoxylin-eosin staining (H&E staining) and observe the alterations under light microscope.

#### 2.17.3 Pathological examination of organs

The collected major mouse organs (liver, kidney, heart, lung, and spleen) were fixed in 4 % paraformaldehyde for 24 h and then stored at −80 °C for further analysis.

### 2.18 Enzyme linked immunosorbent assay

The mice were fasted and treated with water for 12 h the night before the samples were collected. The blood was centrifuged at 3000 rpm for l0 minutes by eye blood collection method to obtain serum, which was stored at −20 °C for later use. A part of the colon tissue was soaked in paraformaldehyde for pathological examination, and the other part of the tissue was minced and added to the saline homogenate, and stored at −80 °C for later use. According to the instruction of ELISA kit, each sample to be tested was added into 96-well plate, and the OD value of each well was measured at 450 nm. Then, the levels of inflammatory factors IL-6, IL-1β and TNF-α were calculated according to the standard curve.

### 2.19 Microbiota analysis

The intestinal microbiota of CRC mice feces was analyzed by 16S rRNA sequencing. First, mice feces were placed in sterile freezing tubes and stored at −80 °C. Then, the DNA was extracted using the TGuide S96 Magnetic Bead Method Soil/Feces Genomic DNA Extraction Kit. Follow the instructions of the kit, the concentration of extracted DNA was detected by enzyme-labeled instrument after extraction. PCR amplification and product purification was performed after detection. PCR quantification was performed with ImageJ software based on the gel electrophoresis results, and the samples were mixed according to the quantitative concentration. Qsep-400 method was used for quality control, and Illumina NovaSeq 6000 was used for on-line sequencing after meeting the specifications. Subsequently, OTU analysis, Alpha diversity analysis, and Beta diversity analysis were conducted using QIIME software. Furthermore, the abundance of intestinal microbiota in each group was analyzed based on the OTU classification results. Frozen fecal samples (50 mg) were mixed with acidified ether (containing 1 % phosphoric acid) and vortex-extracted. After centrifugation, the supernatant was collected and filtered through a 0.22 μm membrane. The concentrations of acetic acid, propionic acid, butyric acid, isobutyric acid, valeric acid, isovaleric acid, hexanoic acid, and isohexanoic acid were quantified using gas chromatography-mass spectrometry (GC-MS, Agilent 7890B/5977A) with an internal standard (deuterated butyric acid). The separation was performed on an HP-FFAP column (30 m × 0.25 mm × 0.25 μm) with helium as the carrier gas (flow rate: 1.0 mL/min).

### 2.20 Immunohistochemical analysis

Immunohistochemical (IHC) analysis of colorectal tumor tissues was performed to detect CD8^+^ T cells (using CD8α antibody), regulatory T cells (FoxP3 antibody), and macrophage polarization phenotypes (pan-macrophage marker CD68; M1/M2 markers CD86 and CD206, respectively). Following heat-induced antigen retrieval, tissue sections were sequentially incubated with primary antibodies (overnight at 4 °C) and HRP-conjugated secondary antibodies for DAB chromogenic development, followed by hematoxylin counterstaining. Immunofluorescence dual-label staining was used to label pan-macrophages, M1, and M2 macrophages with anti-CD68 (Alexa Fluor 594, red), anti-CD86 (Alexa Fluor 488, green), and anti-CD206 (Alexa Fluor 647, blue) antibodies, respectively, with DAPI counterstaining of nuclei and laser confocal microscopy (Zeiss LSM 880).

### 2.21 Statistical analysis

The data for each experimental were analyzed using Graph Pad Prism 9.0 software. All results were presented as “mean ± SD” and comparisons between groups were analyzed by one-way ANOVA. In addition, the *p* values lower than 0.05 (*p* < 0.05) were regarded as statistically significant.

## 3 Results and discussion

### 3.1 Synthesis and characterization of CSM and CM

**Fig. 2:**
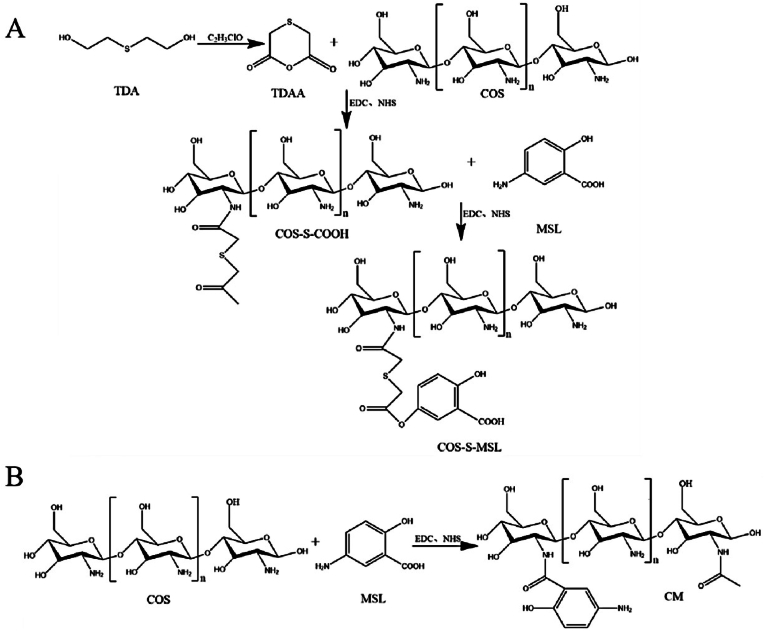
Synthesis process of CSM(A) and CM (B).

For the synthesis of CSM and CM, the synthetic route of CSM was shown in Fig. 2A. Firstly, TDA was dehydrated and condensed to produce anhydride TDAA. Subsequently, TDAA and COS were aminated to produce COS-COOH using DCC and NHS as catalysts. Finally, COS-S-COOH and MSL were similarly amidated to produce COS-S-MSL (CSM). In addition, COS-MSL (CM) was obtained by the amide reaction of COS and MSL, as shown in Fig. 2B.

**Fig. 3:**
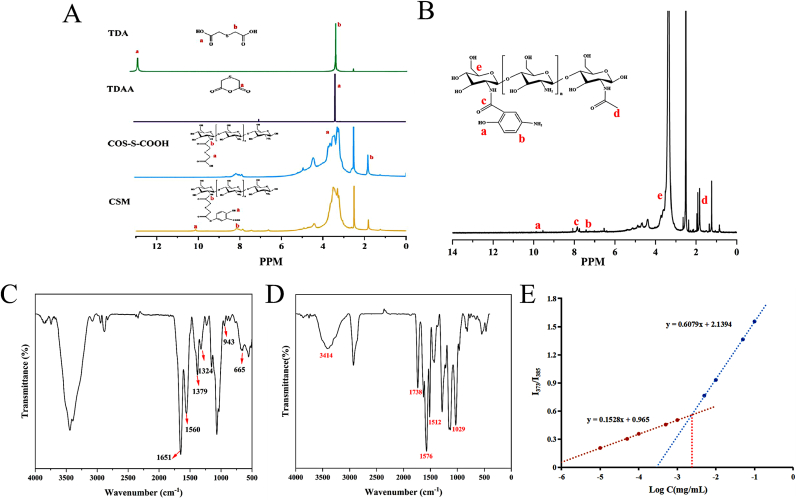
^1^H NMR spectra of TDA, TDAA, COS-S-COOH and CSM (TDA, TDAA, COS-S-COOH and CSM were dissolved in DMSO-*d*_6_ and determined by 400 MHz ^1^H NMR spectrometry) (A). ^1^H NMR spectrum of CM (CM was dissolved in DMSO-*d*_6_ and determined by 400 MHz ^1^H NMR spectrometry) (B). FT-IR spectra of CSM (C) and CM (D). The critical aggregation concentration of CSM(E).

The structural confirmation of CSM and CM polymers was achieved through ^1^H NMR spectroscopy. For the CSM polymer (Fig. 3A), the spectrum revealed critical diagnostic signals: the absence of the carboxyl proton resonance (-COOH) at *δ* = 12.63 ppm in TDAA confirmed successful thiolation, while the appearance of a methylene (-CH_2_-S-CH_2_-) peak at *δ* = 3.64 ppm validated TDAA synthesis. In the COS-S spectrum, resonances within *δ* = 3.23–3.54 ppm corresponded to methylene (-CH_2_-) and methine (-CH-) protons, confirming thiol-functionalized chitosan oligosaccharide formation. The CSM spectrum further exhibited distinct peaks at *δ* = 10.18 ppm (phenolic -OH of MSL), *δ* = 6.64–7.88 ppm (trisubstituted aromatic protons from MSL), and *δ* = 8.14 ppm (amide -NH), collectively confirming covalent conjugation of MSL to the COS-S backbone.

Similarly, the ^1^H NMR spectrum of CM (Fig. 3B) displayed characteristic signals: *δ* = 1.82 ppm (-CH_3_ of acetylated COS) and *δ* = 3.38–3.74 ppm (-CH_2_/-CH of COS backbone) confirmed polysaccharide integrity. Additional peaks at *δ* = 9.71 ppm (phenolic -OH of MSL), *δ* = 6.54–7.73 ppm (aromatic protons of MSL), and *δ* = 7.84 ppm (amide -NH) demonstrated successful MSL grafting. These spectral features conclusively verified the synthesis of both CSM and CM polymers.

Next, FT-IR analysis provided complementary evidence for polymer synthesis. The CSM spectrum (Fig. 3C) exhibited key absorption bands: 665 cm^−1^ (NH_3_^+^ asymmetric bending), 1324 cm^−1^ (C-N stretching), and 943–1379 cm^−1^ (C=O stretching and O-H bending of MSL's carboxyl group). A broad absorption between 3085 and 2552 cm^−1^ corresponded to NH_3_^+^ vibrations on the MSL aromatic ring, while peaks at 1651 cm^−1^ (amide I, C=O) and 1560 cm^−1^ (amide II, N-H) confirmed covalent amide bond formation. Additional MSL-specific bands at 883 cm^−1^ and 808 cm^−1^ (=C-H bending and trisubstituted aromatic ring vibrations) further corroborated successful conjugation.

For CM (Fig. 3D), the spectrum revealed a broad -OH/-NH stretching band at 3414 cm^−1^, C=C aromatic vibrations at 1576 cm^−1^, and O-C alcohol stretching at 1029 cm^−1^. The presence of amide I (1738 cm^−1^, C=O) and amide II (1512 cm^−1^, N-H) bands confirmed MSL attachment, aligning with ^1^H NMR findings.

### 3.2 Determination of the critical aggregation concentration (CAC)

The amphiphilic nature of CSM was quantified by measuring its CAC using pyrene fluorescence. As shown in Fig. 3E, a distinct inflection point at 7.5 × 10^−2^ mg/mL marked the onset of micelle formation, evidenced by a sharp increase in the I_3_/I_1_ fluorescence ratio. This low CAC value underscores the polymer's strong self-assembly propensity under dilute conditions, ensuring micellar stability during systemic circulation. The result provides a foundational parameter for subsequent biocompatibility and drug delivery studies.

### 3.3 Preparation, characterization, and evaluation of PTX/CSM micelles

**Fig. 4:**
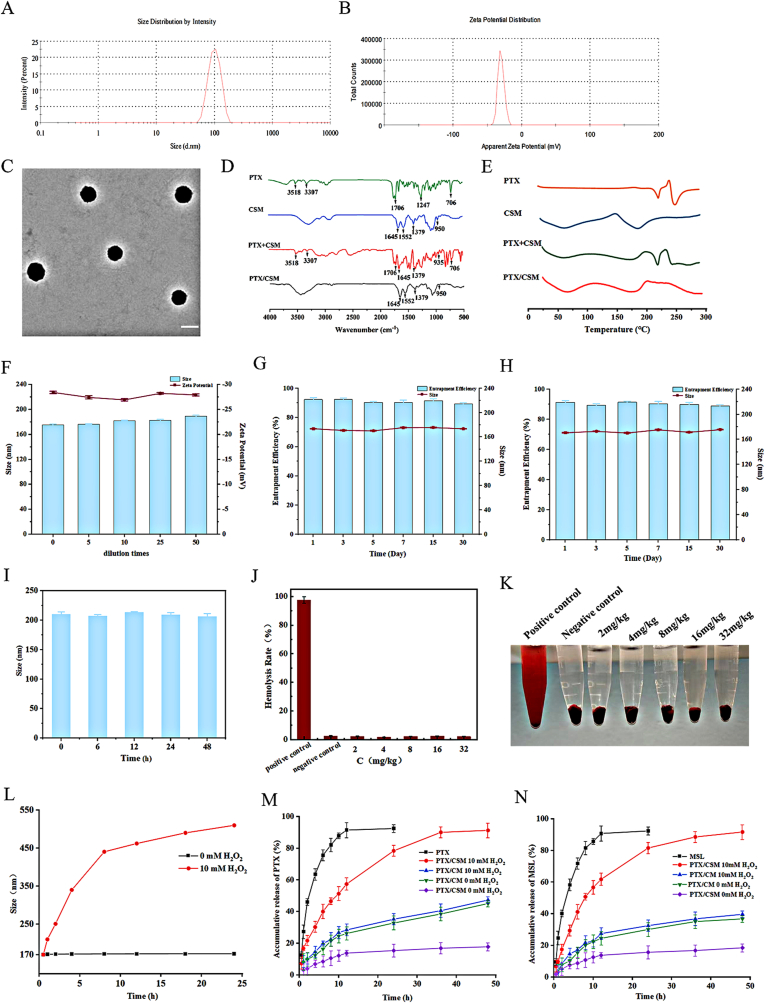
Characterization and stability of PTX/CSM micelles. Hydrodynamic size distribution by DLS (A). Zeta potential distribution (B). TEM micrograph (scale bar: 200 nm) (C). FTIR profiles of PTX/CSM (D). DSC thermograms of PTX, CSM, physical mixture, and PTX/CSM micelles. (E) Dilution stability (0–50 × ). (F–G) Storage stability at 4 °C and 25 °C. (H) Plasma stability. (I–J) Hemolysis assay: visual samples and quantitative hemolysis rates. (K) ROS-triggered size changes. (L–M) *In vitro* cumulative release of PTX and MSL under ROS. (*n* = 3).

PTX/CSM micelles were meticulously developed using the solvent evaporation method, yielding a robust drug delivery system with well-defined characteristics. Initial characterization via DLS demonstrated a monodisperse population, exhibiting an average hydrodynamic diameter of 178.3 ± 0.74 nm, a Zeta potential of −28.4 ± 0.65 mV, and a PDI of 0.171 ± 0.023 (Fig. 4A and B). These metrics signify exceptional colloidal stability, essential for preserving micelle integrity under physiological conditions. Complementing this, TEM revealed spherical micelles with smooth surfaces and uniform size distribution (Fig. 4C, scale bar: 200 nm), attributes that enhance the reliability of drug delivery. The micelles also showcased impressive drug incorporation, achieving a drug loading capacity (DL%) of 8.83 ± 0.78 % and an encapsulation efficiency (EE%) of 92.01 ± 0.20 %. The FTIR spectra of PTX, CSM, physical mixture (PTX + CSM), and PTX/CSM are shown in Fig. 4D. The characteristic peaks of both PTX and CSM can be observed in the physical mixture (PTX + CSM), whereas the characteristic peaks of PTX disappear in PTX/CSM, confirming the successful encapsulation of PTX within the prodrug micelle CSM. DSC further validated successful encapsulation (Fig. 4E), as the characteristic crystalline PTX melting peak at 279.1°C—present in the physical mixture of PTX and CSM—was absent in the micelles, indicating that PTX was effectively amorphized within the micellar core.

Building on their promising structural properties, the stability of PTX/CSM micelles was rigorously assessed to confirm their practical applicability. Dilution stability tests showed no notable size alterations upon 50-fold dilution (Fig. 4F), demonstrating resilience against dilution effects typical during administration. Long-term storage stability was evaluated at 4 °C and 25 °C over 30 days, with the micelles consistently maintaining a PDI below 0.2 (Fig. 4G and H), reflecting their resistance to aggregation or degradation. Furthermore, plasma stability was tested by incubating the micelles in simulated blood conditions (pH 7.4, 37 °C) for 48 h, revealing minimal size changes (Fig. 4I). This stability in plasma underscores their potential for controlled drug release post-intravenous delivery, a critical factor for therapeutic success.

Transitioning to safety considerations, the biocompatibility of PTX/CSM micelles was evaluated through a hemolysis assay across concentrations of 0.1–1 mg/mL. Hemolysis rates remained below 3 % at all levels (Fig. 4J and K), significantly lower than the 100 % hemolysis caused by the positive control. Visual inspection revealed clear supernatants and settled erythrocytes, reinforcing their compatibility with red blood cells. These findings align with pharmacopeial standards for injectable formulations, which require hemolysis rates below 5 %, affirming the micelles’ suitability for intravenous use.

A standout feature of PTX/CSM micelles is their reactive oxygen species (ROS)-responsive drug release mechanism, tailored for tumor-targeted therapy. In the presence of 10 mM H_2_O_2_—mimicking an ROS-rich tumor microenvironment—the micelles exhibited significant disassembly, with particle size escalating from 178 nm to over 400 nm within 24 h (Fig. 4L). In contrast, they remained stable in H_2_O_2_-free phosphate-buffered saline (PBS), confirming ROS-specific triggering. *In vitro* drug release studies illuminated this responsiveness further (Fig. 4M and N). While free PTX and MSL released over 90 % within 24 h, and PTX/CM micelles (non-ROS-sensitive) showed consistent release regardless of H_2_O_2_, PTX/CSM micelles exhibited controlled release in physiological conditions (no H_2_O_2_), with less than 20 % drug release over 48 h. However, in an ROS-rich setting (10 mM H_2_O_2_), release surged to 91.19 ± 1.25 % for PTX and 91.97 ± 2.02 % for MSL within 48 h. This selective release is driven by the oxidative cleavage of thioether and amide bonds in the CSM polymer, enabling precise drug delivery in tumor microenvironments with elevated ROS levels.

In summary, PTX/CSM micelles combine excellent stability, efficient drug encapsulation, and biocompatibility with a sophisticated ROS-responsive release mechanism, positioning them as a highly promising platform for targeted cancer therapy.

### 3.4 Synergistic effect of PTX and MSL at different ratios

**Fig. 5:**
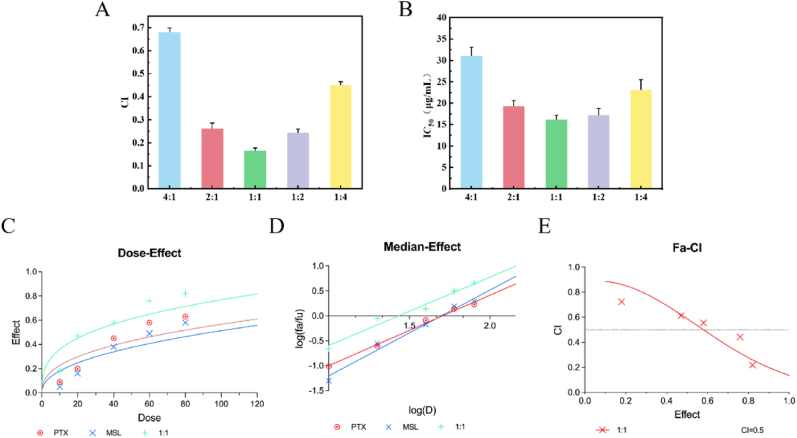
Synergistic interaction analysis of PTX and MSL in SW480/PTX cells. (A) Combination index (CI) values for PTX:MSL molar ratios (1:4 to 4:1) after 48 h co-treatment. Dashed line indicates CI = 1 (additive effect). (B) Corresponding IC_50_ values (mean ± SD, *n* = 3) for PTX:MSL combinations. The 1:1 ratio exhibited the lowest IC_50_ (0.32 ± 0.07 μM). (C) Dose-response curves for PTX, MSL, and their combinations (1:1–4:1). (D) Median-effect plot derived from the Chou-Talalay method. (E) Fraction affected (Fa) vs. CI plot, with CI < 0.3 indicating strong synergism.

The synergistic antiproliferative effects of PTX and MSL on SW480/PTX cells were quantitatively assessed via CCK-8 assay. As illustrated in Fig. 5A and B, combination indices (CI) across all tested PTX:MSL ratios remained below 1.0 after 48 h of co-treatment, demonstrating consistent synergism. Notably, the 1:1 M ratio exhibited the lowest IC_50_ value (0.32 ± 0.07 μM), indicating maximal therapeutic synergy.

The dose-effect curve (Fig. 5C), median-effect plot (Fig. 5D), and Fa-CI curve (Fig. 5E) collectively reinforced the synergistic interaction (CI < 1). Remarkably, CI values within the 2:1 to 1:2 M ratio range fell below 0.3, signifying strong synergism (CI < 0.3: highly synergistic). This pharmacodynamic profile guided the selection of 2:1–1:2 ratios for PTX/CSM micelles formulation, optimizing therapeutic efficacy against paclitaxel-resistant colorectal cancer.

### 3.5 Drug resistance and cytotoxicity in SW480 and SW480/PTX cells

**Fig. 6:**
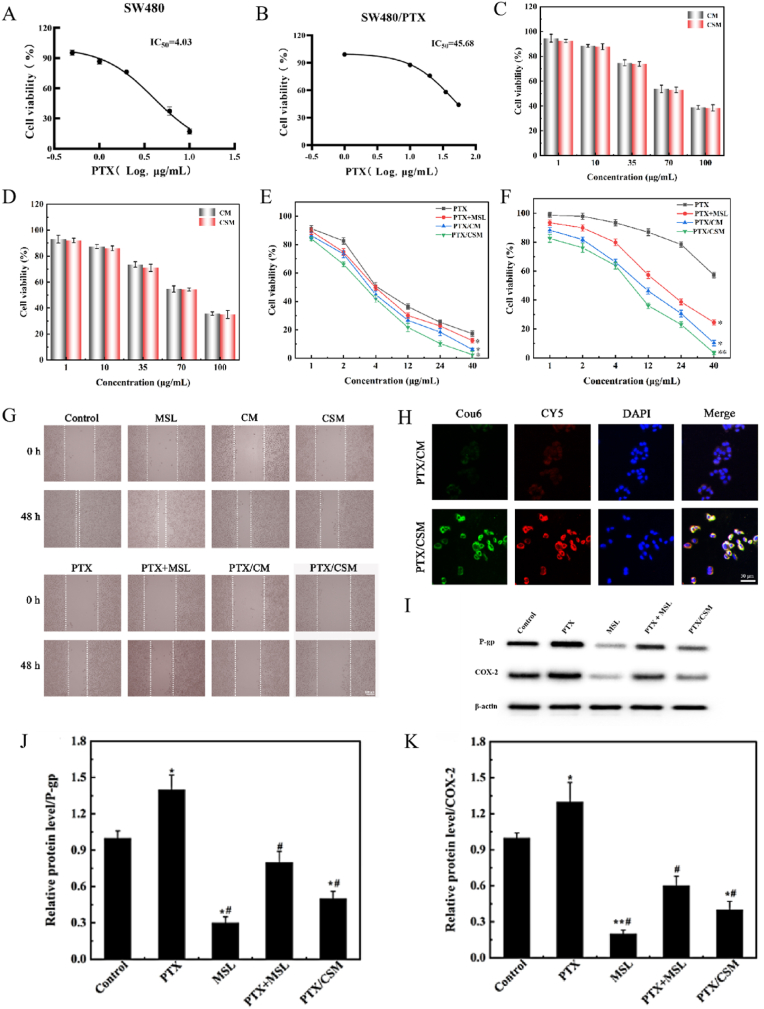
Functional characterization of PTX/CSM micelles in resistant colorectal cancer models. IC_50_ values of PTX on SW480 (A) and SW480/PTX (B) cells (*n* = 3). Effects of cells survival rate of CM and CSM on SW480 (C)and SW480/PTX (D) (*n* = 3). The cells survival rate of different drug groups on SW480 (E)and SW480/PTX cells (F) (*n* = 3, compared with PTX, ∗*p* < 0.05, ∗∗*p* < 0.01). Effects of different treated groups on migration of SW480/PTX cells (G). Cellular uptake of different drug in SW480/PTX cells (scale bars: 50 μm) (H). The protein expression of P-gp in SW480/PTX cells (I). Statistical graph of P-gp (J) and COX-2 (K) protein experimental data (n = 3, ∗*p* < 0.05, ∗∗*p* < 0.01 compared to control group; #*p* < 0.05 compared to model group).

The investigation into drug resistance and cytotoxicity begins with the evaluation of SW480/PTX cells, a paclitaxel-resistant colorectal cancer cell line, using the CCK-8 assay. The IC_50_ values for PTX were determined as 45.68 μg/mL for SW480/PTX cells and 4.03 μg/mL for their sensitive counterpart, SW480 cells (Fig. 6A and B). This disparity yielded a resistance index (RI) of 11.33, well above the threshold of 5, confirming SW480/PTX as a resistant strain. This baseline set the stage for assessing the efficacy of various PTX formulations in overcoming resistance.

**Fig. 7:**
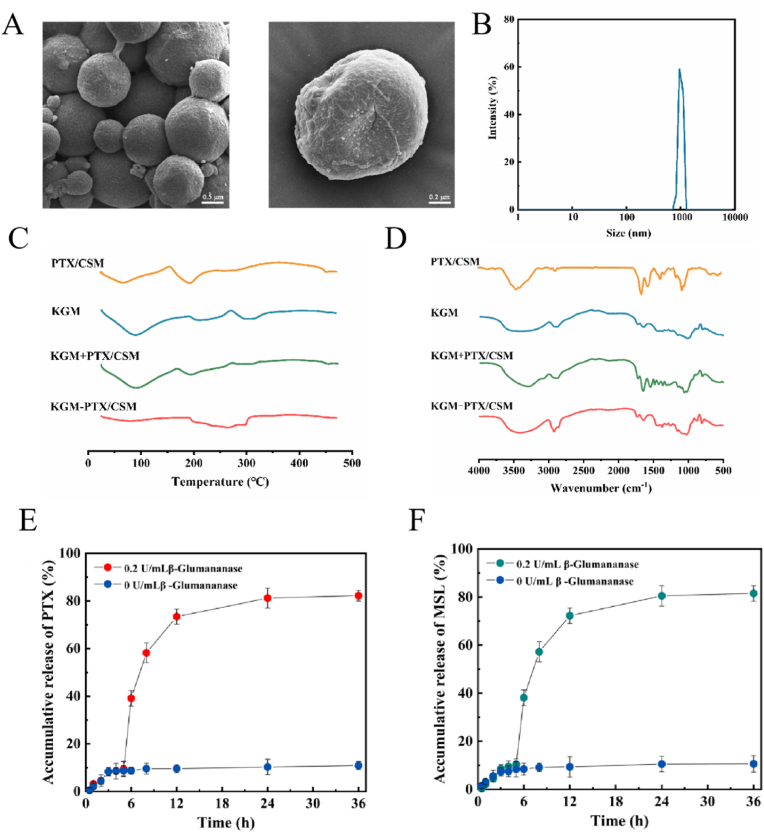
Structural and functional characterization of KGM-PTX/CSM microspheres. (A) Scanning electron microscopy (SEM) image showing the uniform spherical morphology and smooth surfaces of KGM-PTX/CSM microspheres. (B) Particle size distribution of KGM-PTX/CSM microspheres, indicating narrow distribution with a mean diameter of 1.15 ± 0.03 μm and PDI <0.1. (C) DSC profiles comparing KGM-PTX/CSM microspheres with the physical mixture, showing the absence of PTX/CSM characteristic peaks, indicating micellar encapsulation of PTX/CSM. (D) FTIR spectra of KGM-PTX/CSM microspheres, showing the dominance of KGM-specific signatures and the absence of PTX/CSM markers, confirming complete encapsulation. (E) Cumulative drug release profile of PTX from KGM-PTX/CSM microspheres in artificial gastric fluid (pH 1.2), small intestinal fluid (pH 6.8), and artificial colon fluid (pH 7.4 PBS with β-glucomannanase and H_2_O_2_), illustrating controlled release under dual ROS and enzyme sensitivity. (F) Cumulative drug release profile of MSL from KGM-PTX/CSM microspheres, demonstrating similar release kinetics as PTX under the same conditions. Data are expressed as mean ± SD (*n* = 3).

**Fig. 8:**
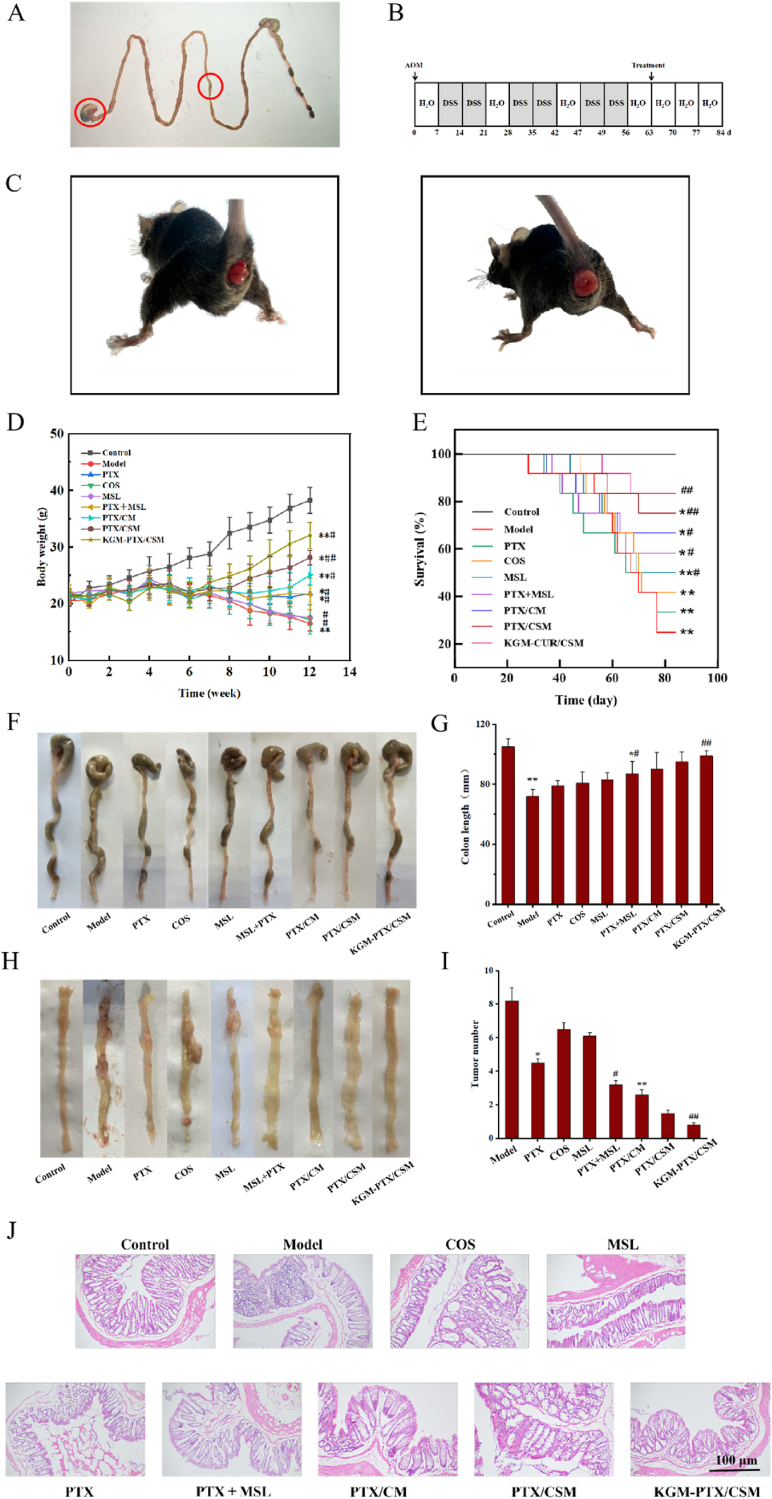
Ex vivo intestinal distribution of IR780 labeled microspheres (A) In *Vivo* Evaluation of Colorectal Cancer (CRC) Mouse Model and Therapeutic Efficacy. Establishment process of the CRC mouse model (B). Prolapse during modeling in model group mice (C). Body weight change curve of mice in each group (D) (*n* = 10, compared with control, ∗*p* < 0.05, ∗∗*p* < 0.01; compared with model, ^#^*p* < 0.05, ^##^*p* < 0.01). Change curve of survival rate of mice in each group (E) (*n* = 10, compared with control, ∗*p* < 0.05, ∗∗*p* < 0.01; compared with model, ^#^*p* < 0.05, ^##^*p* < 0.01). Appearance (F), length distribution (G), physical tumor (H) and distribution of tumor number (I) of CRC mice (*n* = 10, compared with control, ∗*p* < 0.05, ∗∗*p* < 0.01; compared with model, ^#^*p* < 0.05, ^##^*p* < 0.01). Histopathomorphology of CRC mice ( × 100) (J).

Transitioning to cytotoxicity, the blank carriers CM and CSM were tested across both cell lines, revealing concentration-dependent effects (Fig. 6C and D). At ≤50 μg/mL, cell viability exceeded 85 %, indicating low toxicity, whereas at 200 μg/mL, viability fell below 60 %. This drop suggests inherent anticancer properties, likely due to MSL inducing cell cycle arrest and apoptosis, highlighting the carriers' potential for tumor-selective therapy via localized drug accumulation. Building on this, PTX-loaded formulations were evaluated (Fig. 6E and F). Free PTX showed a classic dose-response curve, with SW480/PTX cells exhibiting 11.3-fold greater resistance (IC_50_ = 45.68 ± 1.24 μg/mL) compared to SW480 cells (IC_50_ = 4.03 ± 0.18 μg/mL). Combining free PTX with CSM (1:2 ratio) reduced the IC_50_ in SW480/PTX cells to 17.24 ± 1.23 μg/mL, demonstrating MSL's chemosensitization effect in reversing multidrug resistance.

Encapsulation within micellar carriers further enhanced efficacy. PTX/CSM micelles achieved the lowest IC_50_ values—3.38 ± 0.13 μg/mL in SW480 cells (16 % lower than free PTX) and 9.33 ± 0.18 μg/mL in SW480/PTX cells (4.9-fold better than free PTX)—owing to ROS-triggered disassembly. Elevated intracellular reactive oxygen species (ROS) cleave monosulfide bonds in PTX/CSM, accelerating drug release, whereas PTX/CM micelles, lacking ROS sensitivity, showed an intermediate IC_50_ of 12.24 ± 0.54 μg/mL in SW480/PTX cells. This underscores the role of thioether bonds in tumor-specific activation.

In summary, the PTX/CSM micellar formulation effectively overcomes drug resistance in SW480/PTX cells by integrating MSL-mediated chemosensitization, ROS-responsive drug release, resulting in superior cytotoxicity and intracellular drug accumulation compared to free PTX or less responsive carriers.

### 3.6 PTX/CSM micelles suppress metastatic potential and overcome multidrug resistance in SW480/PTX cells

Beyond cytotoxicity, the antimetastatic potential of PTX formulations was assessed using wound healing assays in SW480/PTX cells (Fig. 6G). The migratory capacity of SW480/PTX cells was assessed through a cell migration assay. After 48 h of observation under a microscope, the control group was basically healed, whereas the cell migratory capacity in all other drug-treated groups was inhibited to a certain extent. Compared to the MSL and CM groups, the CSM group demonstrated a more significant inhibitory effect on the migration of SW480/PTX cells, indicating that CSM was effectively cleaved within the SW480/PTX cells under high ROS conditions, facilitating the intracellular accumulation of MSL. Furthermore, compared to the PTX group, the PTX + MSL, PTX/CM micelles, and PTX/CSM micelles groups displayed significant inhibition of SW480/PTX cell migration. This indicated that the combination of MSL and PTX enhanced SW480/PTX cell sensitivity to PTX, thereby potentiating the inhibitory effect on cell migration. Notably, the PTX/CSM micelles group exhibited the minimal wound healing and the most pronounced growth inhibition among the SW480/PTX cells. This suggests that within the high ROS environment of SW480/PTX cells, the PTX/CSM micelles were efficiently cleaved, resulting in increased drug accumulation and improved therapeutic efficacy.

The uptake process of PTX and MSL by SW480/PTX cells was observed using a fluorescence inverted microscope (Fig. 6H). Both the PTX/CM and PTX/CSM micelles exhibited green and red fluorescence, confirming the successful intracellular release of PTX and MSL in SW480/PTX cells. Additionally, the PTX/CSM micelles with monosulfide bonds demonstrated the most intense intracellular fluorescence. This finding implies that the elevated ROS levels within colorectal tumor cells facilitate the cleavage of these bonds, leading to an increased release of PTX and MSL.

Western blot analysis revealed the key molecular mechanism by which the PTX/CSM nano-prodrug overcomes multidrug resistance in colorectal cancer. As shown in Fig. 6I–K, PTX monotherapy significantly upregulated the expression levels of P-gp and COX-2 in SW480/PTX cells (*p* < 0.01), which activated cellular defense mechanisms under drug-resistant conditions, potentially contributing to multidrug resistance. In contrast, MSL specifically suppressed the expression of P-gp and COX-2 (*p* < 0.01). Mechanistically, MSL inhibited the TNF-α/NF-κB signaling pathway, thereby reducing the expression of inflammation-related drug resistance proteins (e.g, P-gp). As a derivative of 5-aminosalicylic acid (5-ASA), MSL directly inhibited COX-2 activity, decreasing prostaglandin E2 (PGE2) production. Consequently, it interfered with the activation of multiple pro-survival signaling pathways, including c-Jun/AP-1, NF-κB, Akt, and MAPK, while further indirectly suppressing P-gp-mediated drug efflux. Notably, the PTX/CSM nano-prodrug group exhibited the most potent inhibitory effects, reducing P-gp and COX-2 expression levels by approximately 65 % and 70 %, respectively, compared to the PTX monotherapy group (*p* < 0.01). The nanocarrier facilitated stimuli-responsive drug release, enhancing intracellular drug accumulation and promoting the synergistic therapeutic effect of MSL and PTX.

In conclusion, PTX/CSM micelles synergistically inhibit SW480/PTX cell migration and enhanced cellular uptake by leveraging ROS-triggered drug release to enhance intracellular accumulation of PTX and MSL, while dual suppression of P-gp-mediated drug efflux and COX-2-driven inflammatory signaling restores chemosensitivity and amplifies antimetastatic efficacy.

### 3.7 Development and characterization of KGM-PTX/CSM microspheres for colon-targeted drug delivery

The KGM-PTX/CSM microspheres were meticulously developed as an advanced colon-targeted drug delivery system, integrating the chemotherapeutic agent PTX with KGM and PTX/CSM micelles to optimize therapeutic outcomes in colorectal cancer treatment. The preparation and characterization of these microspheres, alongside their dual-responsive drug release behavior, were comprehensively evaluated to ensure their efficacy and specificity.

The microspheres exhibited a uniform spherical morphology, as confirmed by scanning electron microscopy (SEM), with smooth surfaces and robust structural integrity (Fig. 7A). DLS analysis further validated their colloidal stability, revealing a narrow particle size distribution with an average diameter of 1.15 ± 0.03 μm and a PDI of less than 0.1 (Fig. 7B). These properties underscore the consistency and quality of the fabrication process. The system achieved an encapsulation efficiency of 70.83 ± 1.30 % for PTX and a drug loading capacity of 1.73 ± 0.04 %. The slight reduction in these metrics compared to standalone micelles can be attributed to the increased proportion of the KGM carrier matrix in the composite formulation, which enhances structural stability while retaining effective drug incorporation.

Thermal and structural analyses provided robust evidence of successful micellar encapsulation within the KGM matrix. Differential scanning calorimetry (DSC) demonstrated the absence of the characteristic PTX/CSM endothermic peak at 183.29 °C in the microspheres, a feature prominently observed in the physical mixture of KGM and PTX/CSM (Fig. 7C). This disappearance signifies the amorphous dispersion of the micelles within the KGM matrix, confirming effective encapsulation. Fourier-transform infrared (FTIR) spectroscopy further corroborated these findings (Fig. 7D). The FTIR spectrum of the microspheres was dominated by KGM-specific signatures, such as O-H stretching vibrations (3200–3600 cm^−1^) and β-glycosidic linkage peaks (877 cm^−1^), while characteristic PTX/CSM markers—including benzene ring vibrations at 1605 cm^−1^ and amide I bands at 1650 cm^−1^—were completely obscured. This spectral convergence indicates full encapsulation of the PTX/CSM micelles without chemical interaction, ensuring the preservation of both the carrier's structural integrity and the drug's stability during transit through the gastrointestinal tract.

The functional performance of the KGM-PTX/CSM microspheres was assessed through *in vitro* release studies simulating gastrointestinal and colon-specific environments, highlighting their dual sensitivity to ROS and enzymatic activity. Cumulative release curves (Fig. 7E and F) revealed minimal and slow drug release in gastric juice (pH 1.2) and small intestinal fluid (pH 6.8), even in the presence of H_2_O_2_, demonstrating the protective role of the KGM matrix in preventing premature drug liberation. In contrast, drug release was markedly accelerated in artificial colon fluid (pH 7.4) containing β-glucomannanase, particularly under the combined influence of enzymatic activity and ROS. This dual-responsive behavior is driven by a synergistic mechanism: β-glucomannanase hydrolyzes KGM into smaller molecules—such as gluconic acid, monosaccharides, and disaccharides—exposing the encapsulated PTX/CSM micelles, which subsequently undergo ROS-mediated disassembly to release the drug. This targeted release profile ensures precise delivery to the colorectal tumor microenvironment, where both β-glucomannanase and ROS are prevalent.

In conclusion, the KGM-PTX/CSM microspheres represent a sophisticated drug delivery platform, characterized by uniform morphology, stable encapsulation, and a dual-responsive release mechanism tailored for colon-specific therapy. By leveraging the protective and degradable properties of KGM alongside the ROS sensitivity of PTX/CSM micelles, this system minimizes off-target effects and enhances therapeutic efficacy, offering a promising approach for the treatment of colorectal cancer.

### 3.8 In Vivo Evaluation of KGM-PTX/CSM microspheres: gastrointestinal distribution and antitumor efficacy

The gastrointestinal distribution of KGM-PTX/CSM microspheres was investigated using IR780-labeled KGM-PTX/CSM variants to assess their colon-targeting potential. As shown in Fig. 8A, external examination revealed that the green IR780-labeled microspheres were detectable in the stomach and small intestine of mice but remained visually undetectable in the colon. This observation suggests that the microspheres retained their structural integrity throughout the upper gastrointestinal tract, with selective release of IR780 occurring only in the colon. These results highlight the microspheres' excellent colon-targeting efficacy, ensuring that drug release is precisely directed to the intended therapeutic site.

Building on the colon-targeting findings, the therapeutic efficacy of KGM-PTX/CSM microspheres was evaluated *in vivo* using a murine model of CRC induced by AOM and DSS. During the modeling period, daily monitoring of physiological conditions and fecal characteristics revealed distinct differences between groups. Control mice exhibited consistent weight gain, whereas the six treatment groups experienced transient weight loss following AOM injection, followed by a recovery phase. However, subsequent administration of 2 % DSS triggered significant reductions in food intake, body weight loss, fecal abnormalities, and dull fur across these groups. In the second and third DSS cycles, model mice developed severe symptoms, including diarrhea, hematochezia, and statistically significant declines in body weight and food intake (*p* < 0.05). Advanced-stage mice further displayed rectal prolapse, diminished mental status, substantial weight loss, and reduced survival rates. In contrast, the treatment groups exhibited varying degrees of symptom relief, with the KGM-PTX/CSM group showing the most rapid weight recovery and a significantly improved survival rate, as illustrated in Fig. 8D and E.

The progression from colitis to CRC was marked by swelling, ulceration, and thickening of the colorectal mucosa, resulting in a shortened colorectal length and tumor formation. Colorectal length emerged as a reliable indicator of inflammation severity, while tumor number and size reflected CRC progression. Post-modeling analysis of colorectal tissues (Fig. 8F–I) revealed that control mice had smooth, non-erosive colorectums with uniform intestinal walls and no tumors. Conversely, model mice exhibited a significantly reduced colorectal length (*p* < 0.05) and multiple tumors, predominantly in the mid-to-posterior rectum, accompanied by adhesions that complicated dissection. Compared to the model group, the PTX group showed no notable improvement in colorectal length, indicating limited anti-colitis activity, though it achieved a substantial reduction in tumor volume, underscoring PTX's potent anti-CRC effect. The MSL group, on the other hand, demonstrated increased colorectal length, reflecting significant anti-colitis efficacy, alongside a moderate reduction in tumor volume, though less pronounced than in the PTX group. The combination therapy groups—PTX + MSL, PTX/CM, PTX/CSM, and KGM-PTX/CSM—exhibited significant restoration of colorectal length and substantial tumor volume reduction. Among these, the KGM-PTX/CSM group outperformed others, effectively compensating for PTX's limited anti-colitis activity while enhancing the combined anti-CRC effects of PTX and MSL.

To further elucidate the therapeutic impact, histological evaluation of colorectal tissues was conducted using hematoxylin and eosin (HE) staining, as presented in Fig. 8J. The control group displayed normal colorectal morphology with intact epithelial structures. In stark contrast, the model group showed extensive tumor cell infiltration, loss of the epithelial mucus layer, crypts, and goblet cells, widespread inflammation, glandular disarray, enlarged nuclei, and an elevated nucleus-to-cytoplasm ratio. Relative to the model group, the PTX, COS, and MSL groups exhibited partial improvements, including reduced tumor cell presence and partial restoration of tissue architecture. More substantial recovery was observed in the PTX + MSL, PTX/CM, and PTX/CSM groups, characterized by decreased inflammatory cell infiltration, reversal of glandular disorganization, reappearance of goblet cells, and reduced nuclear atypia. Remarkably, the KGM-PTX/CSM group achieved near-complete restoration of colorectal epithelial structure, demonstrating its superior ability to mitigate tissue damage, alleviate cancer-related symptoms, and deliver targeted therapy with combined anti-inflammatory and antitumor effects.

**Fig. 9:**
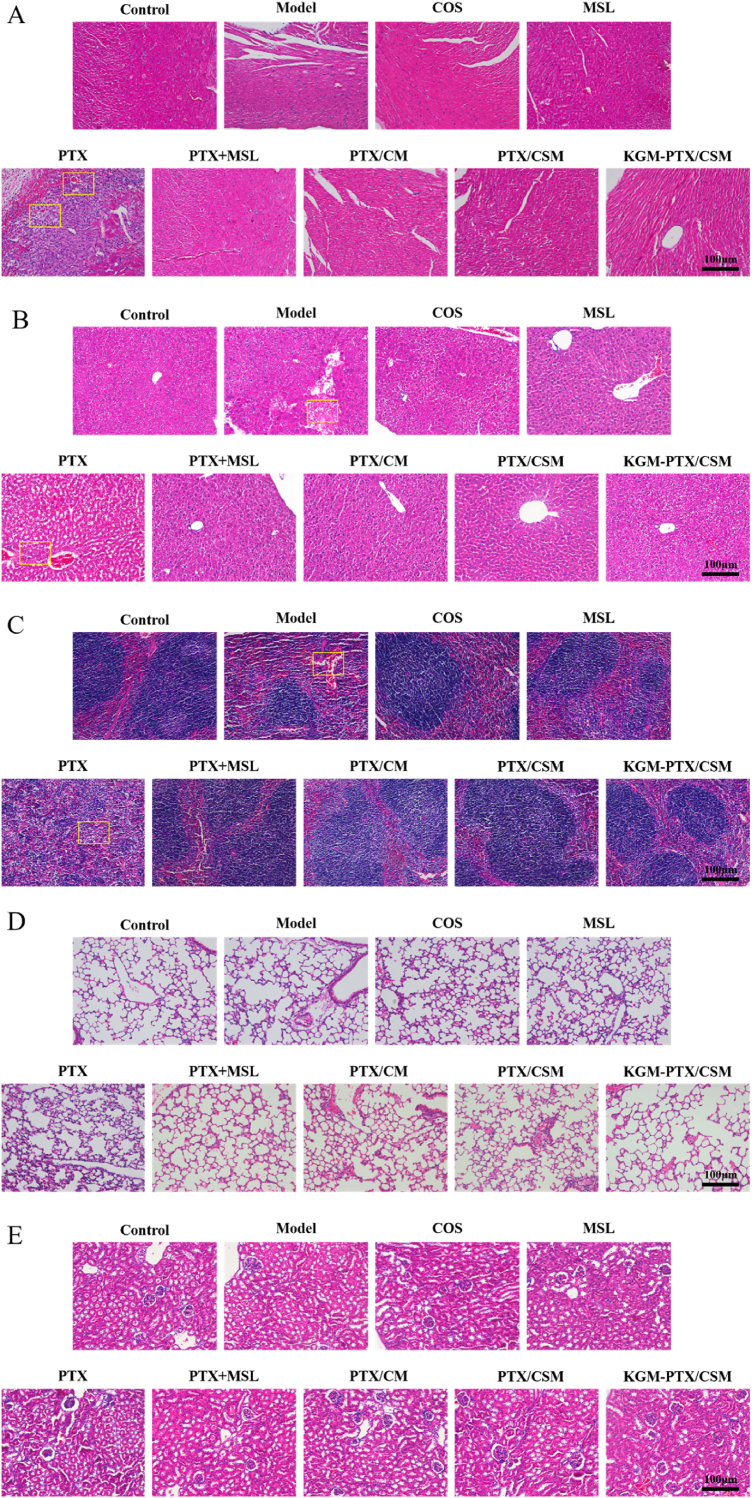
Pathological HE staining results of mouse hearts (A), mouse liver (B), mouse spleen (C), mouse lungs (D) and mouse kidneys (E) ( × 100).

As shown in Fig. 9A–E, the model group exhibited mild hepatic steatosis, inflammatory cell infiltration, and splenic corpuscle atrophy with red pulp congestion, suggesting potential immunosuppression. In the PTX-treated group, focal coagulative necrosis and interstitial fibrosis were observed in cardiac tissue, along with localized inflammatory cell infiltration in hepatic tissue and red pulp fibrosis in the spleen. Other treatment groups showed negligible morphological alterations in murine organ tissues, indicating favorable biosafety profiles. The KGM-PTX/CSM group mitigated acute drug exposure through dual-responsive release kinetics, thereby minimizing systemic PTX toxicity and significantly enhancing drug safety.

In summary, the KGM-PTX/CSM microspheres exhibited exceptional colon-targeting capability, maintaining structural integrity through the upper gastrointestinal tract and selectively releasing their payload in the colon. *In vivo* studies in a CRC mouse model confirmed their therapeutic superiority, with the KGM-PTX/CSM group achieving the most significant improvements in weight recovery, survival rates, colorectal length restoration, and tumor reduction. Histological analysis corroborated these findings, revealing near-complete restoration of colorectal tissue architecture in the KGM-PTX/CSM group. Collectively, these results position KGM-PTX/CSM microspheres as a promising targeted therapy for colorectal cancer, effectively integrating anti-inflammatory and antitumor properties to address both colitis and cancer progression.

### 3.9 Inflammation analysis

**Fig. 10:**
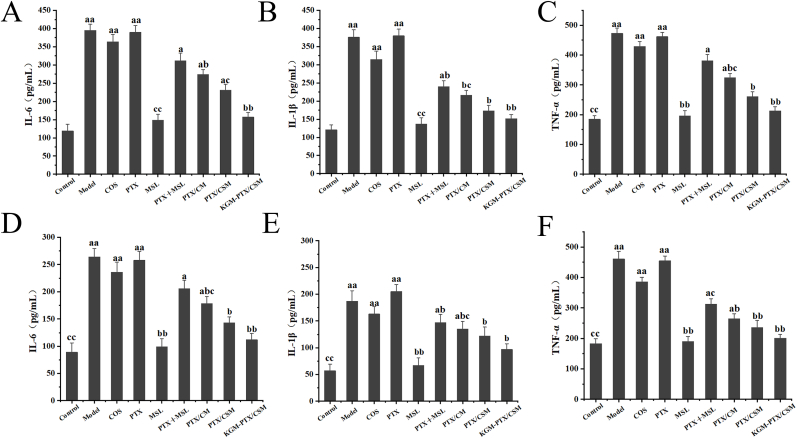
Inflammatory Cytokine Expression in Serum and Colorectal Tissues of CRC Mice. Results of determination of IL-6 (A), IL-1β (B)and TNF-α (C) in mouse serum (*n* = 5, compared with control group, ^a^*p* < 0.05, ^aa^*p*<0.01, compared with model group, ^b^*p* < 0.05, ^bb^*p*<0.01, compared with PTX, ^c^*p* < 0.05, ^cc^*p***<0.01). Results of determination of IL-6 (D), IL-1β (E) and TNF-α (F) in the colorectal tissues of CRC mice (*n*=5, compared with control group, ^a^*p*<0.05, ^aa^*p*<0.01, compared with model group, ^b^*p*<0.05, ^bb^*p*<0.01, compared with PTX, ^c^*p*<0.05, ^cc^*p*<0.01).**

Inflammation is highly associated with the development of colorectal cancer. Anti-inflammatory drugs could enhance the efficacy of anti-colorectal cancer drugs by suppressing inflammatory responses and alleviating the growth and proliferation environment of colorectal tumor cells. Therefore, an enzyme-linked immunosorbent assay (ELISA) was employed to assess the expression levels of inflammatory cytokines, including IL-6, IL-1β and TNF-α, in the serum and colorectal tissues of mice treated with various treatment groups (Fig. 10A–F). The levels of IL-6, IL-1β, and TNF-α in the model group were significantly elevated compared to those in the control group. Compared to the model group, the PTX group also exhibited higher levels in IL-6 and TNF-α, whereas IL-1β content increased. This increase may be attributed to PTX, a known TLR4 ligand that can activate certain cells to release IL-1β, thereby promoting inflammation. The expression levels of IL-6, IL-1β, and TNF-α in the MSL group were all significantly decreased, indicating that MSL possesses favorable anti-inflammatory properties. Compared with the PTX group, the PTX + MSL, PTX/CM, and PTX/CSM groups exhibited significantly reduced levels of IL-6, IL-1β, and TNF-α, mitigating the pro-inflammatory side effects of PTX and effectively inhibiting the expression of inflammatory cytokines. Among these, the PTX/CSM group demonstrated the best therapeutic efficacy, with a notable downregulation of TNF-α, IL-1β, and IL-6 expression in both serum and colorectal tissues. This downregulation was attributed to the presence of reduction-sensitive linkages, thus exhibiting anti-inflammatory effects.

### 3.10 Microbiological analysis of intestinal microbiota in colorectal cancer therapy

Tools like QIIME2 and LEfSe analyze microbial diversity and functional pathways. Multi-omics approaches integrate microbiome and host data to uncover microbial impacts on immune responses and gene expression. These findings support microbiota-based interventions to restore balance and improve CRC therapy. Future research should identify microbial biomarkers and pathways driving CRC for targeted treatments. The development of CRC is often accompanied by gut microbiota dysbiosis. The gut microbiota constitutes a complex microecosystem comprising probiotics (such as Bacteroides, Eubacterium, and Bifidobacterium, which inhibit tumor progression), neutral bacteria, and pathobionts (such as certain strains of Fragilis bacteria, Escherichia coli, etc., which promote tumorigenesis), serving as a crucial component of the CRC microenvironment.

#### 3.10.1 Analysis of the number of OTUs

**Fig. 11:**
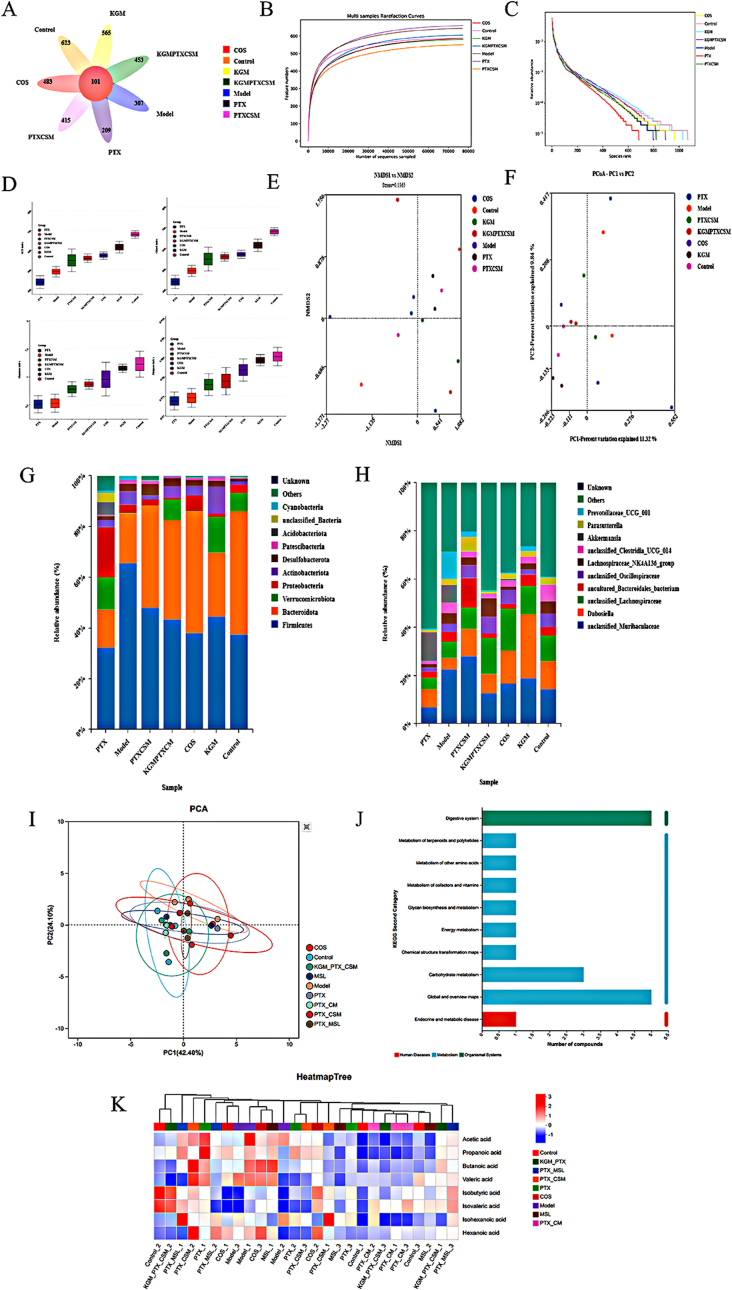
Microbiological Analysis of Gut Microbiota in CRC Mice. Venn diagram of OUT distribution (A), Dilution curves (B) and abundance distribution curves (C). Alpha diversity index (*n* = 3, compared with control, ∗*p* < 0.05, ∗∗*p* < 0.01) (D). Beta diversity analysis(*n* = 3) (E and F). Relative abundance of intestinal microflora (phylum level A (G) and family level B (H)). Analysis of Gut MicrobiotaMetabolism and Short-Chain Fatty Acid Regulation in Different Treatment Groups: PCA (I), KEGG Pathway Enrichment (J), and Heatmap Analyses **(K) (n=3)**.

The Venn diagram in Fig. 11A illustrated the number of shared and unique features among various sample groups, directly depicting the overlap of characteristics between samples. The results indicated that 101 OTUs (operational taxonomic units) were common to the Control, Model, COS, KGM, PTX, PTX/CSM, and KGM-PTX/CSM groups. The unique OTU counts for these groups were 623, 307, 483, 565, 209, 415, and 453, respectively. Compared to the Control group, the Model group exhibited a significant decline in gut microbiota abundance, indicating that the CRC is accompanied by gut microbiota dysbiosis, decreased microbiota abundance, and reduced diversity. When compared to the Model group, the PTX group also showed a reduction in microbiota count, suggesting that PTX may disrupt the gut microbiota during CRC treatment, potentially indirectly promoting tumor progression and compromising its anticancer efficacy. In contrast, the increased microbiota counts observed in the COS and KGM groups indicated that these compounds possessed the capacity to restore gut microbiota balance, thereby inhibiting the progression of the CRC microenvironment and enhancing anticancer effects. Notably, in comparison to the PTX group, the KGM-PTX/CSM group exhibited a significantly higher abundance of gut microbiota, suggesting that COS and KGM could mitigate the gut microbiota dysbiosis of the gut microbiota caused by PTX, mitigate its toxicity and side effects, and indirectly enhanced its therapeutic outcomes.

#### 3.10.2 Analysis of dilution and abundance distribution curve

Dilution curves were employed to assess the coverage of sequencing volume to taxa, reflecting species richness and diversity. Fig. 11B and C showed that the observed plateauing of the curves as the sequencing volume increases, indicating adequate sequencing for analysis. The abundance distribution curve displayed species richness and evenness, and the broad and flat curve indicative of high species richness and a uniform distribution across taxa. Compared with model group, the curve of PTX group was shorter, indicating that gut microbiota was damaged. The curves of COS and KGM groups were long, indicating that they could repair the microbiota and improve the abundance. Notably, the KGM-PTX/CSM group showed a marked improvement in gut microbiota composition relative to the PTX group alone, indicating that COS and KGM could alleviate the disruption caused by PTX to gut microbiota, thereby reducing its toxic and side effects, and enhanced the therapeutic efficacy of PTX when used in combination.

#### 3.10.3 Analysis of alpha diversity

The Chao1 and Ace indices in Alpha diversity (Alpha diversity) were adopted to assess species richness, and the Shannon and Simpson indices were used to indicate the diversity of species, and the trends of these indices showed consistency among the groups (Fig. 11D). Compared to the Control group, the Model group demonstrated significant decreases in the Chao1, Ace, Shannon, and Simpson indices, which suggested that colorectal cancer not only disrupted the balance of intestinal microbiota, reducing the richness of fundamental bacterial species, but also changed the composition of intestinal microbiota, suppressing the development of microbial diversity. Compared with the Model group, PTX group presented a downward trend in all four indices of Alpha diversity, suggesting that PTX may be accompanied by the destruction of intestinal microbiota during the treatment of colorectal cancer. However, the diversity and richness of intestinal microbiota in the KGM-PTX/CSM group were significantly higher than those in the PTX group, suggesting that the synergistic effect of COS and KGM enhanced the diversity of intestinal microbiota and improved the structural composition of the microbiota, thereby strenngthening the overall anti-inflammatory and anti-colorectal cancer effects.

#### 3.10.4 Analysis of beta diversity

Beta diversity analysis could reveal differences in the distribution of species abundance between groups and was an effective approach of comparing differences between ecosystems. PCA analysis (Fig. 11E and F) indicated that a closer distance between data points of two samples implies a greater similarity in their composition. The data points for the PTX group and the Model group were adjacent, while those for the KGM group, COS group, PTX/CSM group, KGM-PTX/CSM group, and control group clustered together. This suggested that the microbial community structures of the PTX group and the Model group were similar, indicating that PTX did not restore the disruption of intestinal microbiota caused by colorectal cancer. In contrast, COS and KGM exhibited significant ameliorative effects on intestinal microbiota, with their community compositions largely resembling that of the control group. This implied that the KGM-PTX/CSM group exhibited superior anti-colorectal cancer efficacy. Furthermore, NMDS analysis, an indirect gradient analysis method based on dissimilarity matrices or distance matrices for ordering, yielded a Stress value of 0.1537 (less than 0.2, indicating that the Stress value reflects the discrepancy between the distances in the reduced-dimensional space and the original multidimensional space). This indicated that the analysis results possessed high credibility and were capable of clearly separating different intestinal microbiota compositions.

#### 3.10.5 Analysis of intestinal microbiota structure in CRC mice

The impact of KGM-PTX/CSM on intestinal microbiota composition was examined by assessing the distribution of predominant phyla and families across the treatment groups (Fig. 11G and H). The gut microbiota composition at the phylum level was dominated by Firmicutes, Bacteroidota, Deferribacterota, Proteobacteria, and Desulfobacterota. Firmicutes was the most abundant group of bacteria in the gut, possessing a variety of physiological functions. Bacteroidota, another significant phylum, contributes to digestion, immune regulation, and nutrient absorption. The ratio between Firmicutes and Bacteroidota (F/B ratio) was often used as one of the indicators to assess gut health status. When this ratio was imbalanced, it may lead to intestinal inflammation. Desulfobacterota was associated with gut microbiota dysbiosis and mild intestinal inflammation. Compared to the Control group, the proportion of Firmicutes increased in both the Model and PTX groups, while the Bacteroidota abundance decreased by approximately 30 % in both groups. Consequently, the F/B ratio increased by 4-fold and 2-fold, respectively, indicating a disruption of the gut microbiota in the Model and PTX group mice, which may lead to metabolic disturbances and inflammation. Notably, the proportion of Desulfobacterota significantly increased in the PTX group, suggesting that PTX disrupted the gut microbiota and indirectly contributed to the development of colorectal inflammation. In contrast, the KGM-PTX/CSM treatment group normalized the gut microbiota structure by reducing the abundance of Firmicutes and increasing that of Bacteroidota, thereby restoring the F/B ratio to normal levels. Additionally, KGM enhanced the abundance of Verrucomicrobia, while COS decreased the abundance of Actinobacteria. Collectively, the KGM-PTX/CSM treatment group, leveraging the synergistic effects of COS and KGM, mitigated the gut microbiota disruption induced by PTX, potentially enhancing its therapeutic efficacy against colorectal cancer.

The composition of the gut microbiota was primarily dominated by families such as Lachnospiraceae, Muribaculaceae, Prevotellaceae, and Bacteroidaceae. Lachnospiraceae was associated with the metabolism of the host, capable of producing multiple short-chain fatty acids that inhibited the development of colorectal cancer. Additionally, unclassified-Muribaculaceae could suppress the growth and reproduction of harmful and putrefactive bacteria in the intestine, thereby improving intestinal disorders. Furthermore, it produced extracellular soluble polysaccharides that exhibited antitumor properties. Compared to the Control group, the proportions of Lachnospiraceae and unclassified-Muribaculaceae significantly decreased in both the Model and PTX groups, indicating disruption in the equilibrium of beneficial bacteria and an imbalance in the gut microbiota. This dysbiosis may indirectly promote the development of colorectal cancer. In contrast, the KGM-PTX/CSM group exhibited a significant increase in the relative abundance of Lachnospiraceae and unclassified-Muribaculaceae compared to the model and PTX groups. (Lachnospiraceae and unclassified-Muribaculaceae) caused by PTX, potentially enhancing the therapeutic efficacy against colorectal cancer by restoring the gut microbiota balance.

#### 3.10.6 Determination results of short-chain fatty acids (SCFAs)

As shown in Fig. 11I–K, a significant reduction in all measured SCFAs (acetic, propionic, and butyric acids) was observed in the model group, consistent with chemotherapy - induced gut dysbiosis. The PTX group exhibited low levels of SCFAs, with butyric acid showing the most pronounced reduction, further confirming that PTX treatment disrupts the gut microbiota, reducing the abundance of SCFA - producing bacteria. In contrast, the COS group showed a significant increase in SCFA levels, with propionic acid exhibiting the most notable elevation due to the promotion of beneficial bacteria like Bifidobacteria. The MSL group demonstrated a general trend of improved SCFA levels compared to the model group, although specific levels were not explicitly detailed. The PTX + MSL group showed higher SCFA levels than the PTX group, suggesting a synergistic effect in promoting beneficial bacteria growth. The non - sensitive prodrug group had higher SCFA levels than the PTX group but lower than the sensitive prodrug and microsphere groups. The sensitive prodrug group exhibited significantly elevated SCFA levels, with butyric acid levels notably higher than in the non - sensitive prodrug group. Most remarkably, the KGM - PTX/CSM group showed the highest levels of all SCFAs. The colon-targeted release of KGM-PTX/CSM ensured β-mannanase-dependent degradation of KGM in the colon, generating β-mannooligosaccharides that selectively stimulate butyrate-producing bacteria (e.g., Roseburia spp.). Elevated butyrate further enhanced PTX's cytotoxicity by downregulating P-gp expression via HDAC inhibition, as evidenced by the reversal of PTX resistance in SW480/PTX cells.

The restoration of butyrate by KGM-PTX/CSM provided a critical link between gut microbiota modulation and antitumor efficacy. Butyrate, a microbial metabolite with dual HDAC inhibitory and immunomodulatory properties, enhances PTX-induced apoptosis while reprogramming the tumor immune microenvironment. This is evidenced by increased CD8^+^ T cell infiltration, reduced Tregs, and M1 macrophage polarization. Notably, butyrate's ability to suppress P-gp expression aligns with *in vitro* data showing reversal of PTX resistance, underscoring the translational potential of targeting the microbiota-metabolite-immune axis in CRC therapy. Furthermore, SCFAs collectively inhibit pro-inflammatory cytokines such as IL-6 and TNF-α, thereby reducing chronic inflammation that is intricately linked to CRC progression. By blocking the NF-κB/STAT3 pathway, SCFAs reduce the release of pro-inflammatory factors and CRC cell proliferation. KGM-PTX/CSM microspheres effectively modulated the gut microbiota, enhance SCFA production, and reprogram the tumor immune microenvironment, thereby exerting a multi-faceted anti-CRC effect through the microbiota-metabolite-immune axis.

### 3.11 Results of immunohistoanalysis

**Fig. 12:**
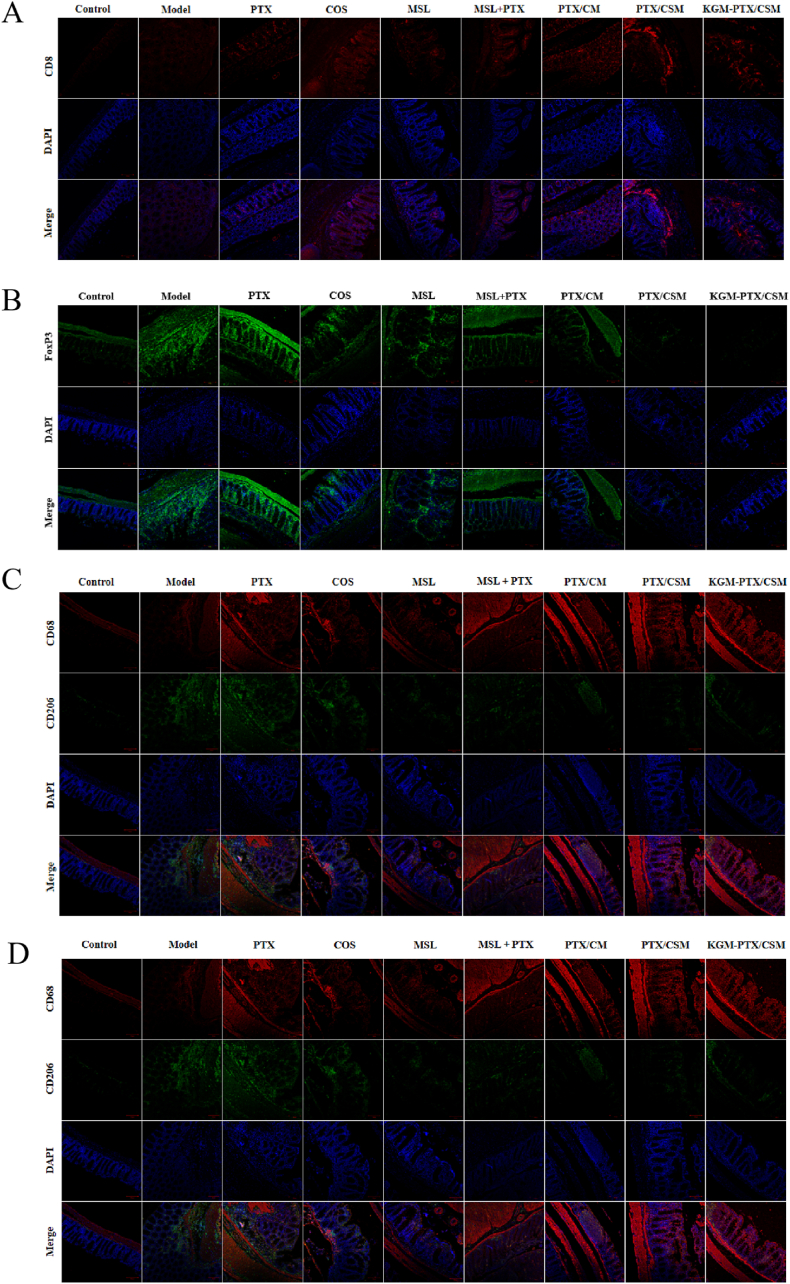
CD8^+^ T Cell Infiltration in Colorectal Tumor Microenvironment (scale bars: 100 μm), CD8^+^ (green), DAPI (blue), merged channels (A). FoxP3+ Regulatory T Cell (Treg) Accumulation (scale bars: 100 μm). FoxP3 (red), DAPI (blue), merged channels (B). Macrophage polarization dynamics: M1 (CD68^+^) vs. M2 (CD206+) phenotype modulation(scale bars: 100 μm). CD68 (total macrophages, green), CD206 (M2 marker, red), DAPI (blue), merged channels (C). Co-localization analysis of M1-polarized macrophages and immune checkpoints (scale bars: 100 μm). CD68 (total macrophages, green), CD86 (M1 marker, red), DAPI (blue), merged channels (D). (For interpretation of the references to color in this figure legend, the reader is referred to the Web version of this article.)

CD8^+^ T cells are crucial for anti-tumor immunity. As shown in Fig. 12, the density of CD8+T cells was significantly increased in the KGM-PTX/CSM group compared to other groups. The model group had low CD8+T cell density, indicating immune evasion in the tumor microenvironment. While the PTX group showed a slight increase in CD8+T cells due to chemotherapy-induced immunogenic cell death, the MSL group demonstrated a moderate rise attributed to reduced immune suppression from anti-inflammatory effects. The COS group exhibited a marked increase in CD8+T cells, likely due to the immunostimulatory properties of chitosan oligosaccharides (COS) in activating dendritic cells. The PTX + MSL combination group showed a synergistic effect, with higher CD8+T cell density than individual drug treatments.

Tregs are known to suppress the function of effector T cells, promoting tumor immune evasion. As shown in Fig. 12A, the model group showed a high proportion of FoxP3+Tregs, indicative of an immunosuppressive tumor microenvironment. While the PTX group showed a slight decrease in Treg density due to the cytotoxic effects of chemotherapy on immune cells, the MSL group exhibited a more pronounced reduction. This is likely attributed to MSL's anti-inflammatory effects in decreasing the recruitment of Tregs by inhibiting pro-inflammatory signals and immune cell expansion. The COS group also demonstrated a decrease in Tregs, possibly through gut microbiota regulation to suppress immune inhibition. The KGM-PTX/CSM group demonstrated the lowest Treg proportion, indicating the combined effects of multiple mechanisms in suppressing immunosuppressive cells and potentially enhancing the efficacy of anti-tumor immune responses.

In macrophage polarization (M1/M2 Phenotype) immunofluorescence analysis results (Fig. 12B and C), the CD68^+^ macrophage density of model group was high, with a predominance of the M2 phenotype (CD206), indicative of an immunosuppressive tumor microenvironment favoring tumor growth. The PTX group showed a potential shift in macrophage polarization, with decreased M2 markers (CD206) and increased M1 markers (CD86), possibly through the inhibition of STAT3 signaling by PTX. This reduces M2-associated gene expression and promotes the secretion of pro-inflammatory factors like IL-6 and TNF-α, steering macrophages toward an M1 phenotype. The MSL group, by inhibiting the NF-κB pathway and reducing the secretion of pro-inflammatory factors such as IL-6 and IL-1β, demonstrated a trend toward decreased M1 macrophage activation [44]. In contrast, the COS group showed enhanced M2 functionality, as certain polysaccharides like COS can activate the PI3K/Akt pathway, increasing IL-10 secretion and supporting anti-inflammatory and tissue repair processes. Most importantly, the KGM-PTX/CSM group exhibited a significant increase in the M1/M2 ratio (CD86+↑, CD206+↓), indicating that the microparticles effectively promote the polarization of macrophages toward an antitumor phenotype by integrating colon-specific delivery, ROS-responsive drug release, and gut microbiota regulation.

As shown in Fig. 12D, the model group exhibited weak CD86^+^ signaling with dominant M2-type (CD206+) macrophages, reflecting an immunosuppressive tumor microenvironment. In the PTX group, CD86^+^ signals were partially enhanced, but co-localization appeared dispersed, suggesting that although the chemotherapeutic agent induced partial M1 polarization, its non-targeted delivery limited therapeutic efficacy. The PTX/CSM group demonstrated superior CD86^+^ signal intensity and co-localization compared to PTX alone, highlighting the advantage of ROS-responsive drug release in localized microenvironment modulation. Following KGM-PTX/CSM treatment, significant activation of M1 macrophages and increased CD8+T-cell infiltration were observed. The polarized M1 macrophages released pro-inflammatory cytokines (e.g., IL-12, TNF-α), which activated CD8+T cells, enhancing their proliferation and cytotoxic function. Concurrently, upregulated MHC-II expression improved tumor antigen presentation, synergizing with T-cell responses. These effects aligned with observed Treg reduction, collectively alleviating immunosuppression. Furthermore, this treatment group elevated short-chain fatty acid levels (e.g., butyrate), directly promoting M1 polarization while indirectly suppressing PD-L1-mediated immune evasion.

Collectively, KGM-PTX/CSM therapy drives a robust shift toward pro-inflammatory/anti-tumor M1 phenotypes, establishing a dynamic network of "immune activation–immunosuppression relief–metabolic reprogramming." These findings demonstrate that the KGM-PTX/CSM system orchestrates multi-mechanistic synergy to remodel the immunosuppressive tumor microenvironment.

## 4 Conclusion

This study was designed to address critical challenges in colorectal cancer treatment, including low drug delivery efficiency, strong drug resistance, and intestinal microenvironment imbalance. A ROS/enzyme dual-responsive cooperative drug delivery system was innovatively constructed to achieve organic integration of precision therapy and multi-mechanism regulation. The amphiphilic prodrug polymer CSM was synthesized by conjugating COS with MSL through thioether bonds, which endowed the system with both ROS-responsive properties and dual drug/carrier functionality. Highly stable PTX/CSM micelles with exceptional drug loading capacity were successfully prepared. Furthermore, by utilizing the colon-targeting capability and enzyme-responsive characteristics of konjac glucomannan, KGM-PTX/CSM composite microspheres were developed to establish a dual precision delivery mode, enabling enzyme-controlled targeted accumulation in the colon followed by ROS-triggered drug release in the tumor microenvironment. The system broke through the limitations of the single response mechanism of the traditional drug delivery system, and significantly improved the penetration and enrichment efficiency of drugs in the lesion site through the collaborative innovation of prodrug functionalization design, vector-targeted modification and intelligent drug release strategy, providing a new functional delivery platform for the treatment of CRC.

The study revealed a multidimensional cooperative antitumor mechanism involving chemosensitization–microbiota remodeling–immune regulation. The CSM prodrug micelles were found to reverse tumor cell drug resistance by downregulating the COX-2/P-gp pathway, thereby enhancing paclitaxel sensitivity and suppressing cell migration. Meanwhile, MSL was demonstrated to significantly ameliorate the tumor inflammatory microenvironment by inhibiting pro-inflammatory factors (e.g., TNF-α, IL-6). The KGM microspheres enabled colon-specific release via enzyme responsiveness, while their prebiotic properties were observed to (i) restore gut microbiota structure, (ii) promote beneficial bacterial proliferation, (iii) reduce pro-inflammatory cytokine levels, and (iv) drive macrophage polarization toward the antitumor M1 phenotype, ultimately enhancing CD8+T cell activity. This system integrates multiple functions, including controlled drug release, drug resistance reversal, anti-inflammatory modulation, microbiota remodeling, and immune activation, thereby establishing a dynamic therapeutic network that combines targeted delivery-microenvironment remodeling–multimechanism synergy. Compared to conventional therapies, this strategy not only enhances anti-CRC efficacy but also mitigates chemotherapy-associated side effects, providing a highly effective and low-toxicity integrated solution for colorectal cancer treatment.

## CRediT authorship contribution statement

**Weitong Sun:** Writing – original draft, Methodology, Investigation, Conceptualization. **Bingbing Fan:** Writing – original draft, Software, Methodology. **Xiaohan Qin:** Software, Resources, Methodology, Conceptualization. **Xin Zhang:** Writing – original draft, Software, Resources, Methodology. **Pengxia Zhang:** Resources, Formal analysis, Data curation. **Yu Zhang:** Writing – review & editing, Supervision, Software, Project administration, Data curation.

## Funding

This work was supported by the Heilongjiang Province Higher Education Excellent Innovation Team Basic Research Fund [grant numbers 2023-KYYWF-0640]; 10.13039/501100005046Natural Science Foundation of Heilongjiang Province of China [grant numbers LH2022H095]; the Heilongjiang Postdoctoral Foundation [grant numbers LBH-Z21214]; the Heilongjiang Provincial Key Laboratory of New Drug Development, the Pharmacotoxicological Evaluation [grant numbers KFKT2021-07] and North Medicine and Functional Food Characteristic Subject Project of Heilongjiang Province [grant numbers No. HLJTSXK-2022-03].

## Declaration of competing interest

The authors declare that they have no known competing financial interests or personal relationships that could have appeared to influence the work reported in this paper.

## Data Availability

Data will be made available on request.
